# A methodical exploration of imaging modalities from dataset to detection through machine learning paradigms in prominent lung disease diagnosis: a review

**DOI:** 10.1186/s12880-024-01192-w

**Published:** 2024-02-01

**Authors:** Sunil Kumar, Harish Kumar, Gyanendra Kumar, Shailendra Pratap Singh, Anchit Bijalwan, Manoj Diwakar

**Affiliations:** 1 Department of Computer Engineering, J. C. Bose University of Science and Technology, YMCA, Faridabad, India; 2 Department of Information Technology, School of Engineering and Technology (UIET), CSJM University, Kanpur, India; 3 Department of Computer and Communication Engineering, Manipal University Jaipur, Jaipur, India; 4 School of Computer Engineering and Technology, Bennet University, Greater Noida, India; 5 Faculty of Electrical and Computer Engineering, Arba Minch University, Arba Minch, Ethiopia; 6 Department of Computer Science and Engineering, Graphic Era Deemed to Be University, Dehradun, India

**Keywords:** CNN, CT scan, Deep learning, Ensemble learning, Transfer learning, X-ray

## Abstract

**Background:**

Lung diseases, both infectious and non-infectious, are the most prevalent cause of mortality overall in the world. Medical research has identified pneumonia, lung cancer, and Corona Virus Disease 2019 (COVID-19) as prominent lung diseases prioritized over others. Imaging modalities, including X-rays, computer tomography (CT) scans, magnetic resonance imaging (MRIs), positron emission tomography (PET) scans, and others, are primarily employed in medical assessments because they provide computed data that can be utilized as input datasets for computer-assisted diagnostic systems. Imaging datasets are used to develop and evaluate machine learning (ML) methods to analyze and predict prominent lung diseases.

**Objective:**

This review analyzes ML paradigms, imaging modalities' utilization, and recent developments for prominent lung diseases. Furthermore, the research also explores various datasets available publically that are being used for prominent lung diseases.

**Methods:**

The well-known databases of academic studies that have been subjected to peer review, namely ScienceDirect, arXiv, IEEE Xplore, MDPI, and many more, were used for the search of relevant articles. Applied keywords and combinations used to search procedures with primary considerations for review, such as pneumonia, lung cancer, COVID-19, various imaging modalities, ML, convolutional neural networks (CNNs), transfer learning, and ensemble learning.

**Results:**

This research finding indicates that X-ray datasets are preferred for detecting pneumonia, while CT scan datasets are predominantly favored for detecting lung cancer. Furthermore, in COVID-19 detection, X-ray datasets are prioritized over CT scan datasets. The analysis reveals that X-rays and CT scans have surpassed all other imaging techniques. It has been observed that using CNNs yields a high degree of accuracy and practicability in identifying prominent lung diseases. Transfer learning and ensemble learning are complementary techniques to CNNs to facilitate analysis. Furthermore, accuracy is the most favored metric for assessment.

## Introduction

Lung diseases are conditions classified as medically aberrant and impair the functionality of the lungs. Typically, the medically abnormal status of the lung is accompanied by a few specific signs and symptoms. Some intrinsic malfunction of the lungs stimulates the progression of the diseases. The World Health Organization (WHO) reported the top ten fatal diseases from 2000 to 2019. Unexpectedly, the majority of these are lung-related, including COPD ranking third, lower respiratory infections ranking fourth, and trachea, bronchus, and lung cancer ranking sixth in mortality causes [1]. Among the ailments that affect the lower respiratory tract, the most common ones are pneumonia, bronchitis, and influenza [2]. Chronic respiratory diseases (CRDs) are incurable conditions that disrupt the delicate balance of the lungs. They mainly appear as COPD and asthma-causing impairments.

Surprisingly, most deaths related to COPD occur in people under 70 years old. The impact is striking, with COPD claiming about 3 million lives yearly, accounting for 6% of mortality. Asthma is also widespread, affecting children and adults, with around 262 million individuals affected [3]. We will never forget the pandemic kind of lung disease that we live with, known as the novel COVID-19, caused by the SARS-CoV-2 virus. As of 2023, the WHO estimates that the virus has infected over 663 million individuals and generated around 7 million fatalities [4]. A considerable number of people die worldwide as a result of lung diseases and their various prominent forms.

Traditional diagnostic procedures focus on manual symptom analysis to diagnose lung illnesses, with clinicians directing future prescription selections based on disease features evaluated [5]. However, the Association of Interdisciplinary Fields causes technology to be coupled with manual analysis for computer-aided diagnosis. As a result, the healthcare sector relies on technology such as medical imaging and ML. Medical imaging refers to the techniques and technologies used to produce visual representations of the interior of a body. In recent years, it has been widely applied to healthcare. It plays a significant role in modern medicine and is used in almost every aspect of patient care, such as diagnosis, therapy, and surgery. It helps clinicians identify and pinpoint disease progressions more precisely. Numerous imaging modalities have been utilized to detect and analyze lung diseases, including chest X-rays [3], CT scans [6], MRI [7], PET [6], sputum smear microscopy images (SSMI) [8], and molecular imaging [9]. X-rays and CT scans are the most commonly used anatomic imaging modalities for detecting and diagnosing various lung diseases [6].

ML has significantly impacted medical imaging, and there has been substantial progress in applying ML-based detection approaches and algorithms. ML can diagnose lung disorders using images from medical or radiological procedures [10]. ML, a subfield of artificial intelligence (AI), tries to make computers learn from data [11]. Consequently, ML offers an automated framework that may be utilized to detect or anticipate lung illnesses in their earliest stages compared to manual methods [12].

Identifying prominent lung conditions such as Pneumonia, Lung cancer, and COVID-19 using imaging and ML encounters some impediments:

- The intricate characteristics of lung structures and the overlapping patterns of diseases might result in misinterpretations.
- Various imaging methods may lead to differences in the quality and consistency of data.
- The scarcity of labeled datasets impeded the training of accurate models, particularly regarding rare illnesses.
- The progressive characteristics of disorders such as COVID-19 provide difficulty for pre-existing models.
- Some solutions can be opted to deal with these impediments:
- Model generalization may be improved by supplementing datasets with diversified samples and assuring uniform imaging techniques.
- Continuous model adaption via real-time data updates is critical, particularly with changing features.
- Using ML approaches may improve model interpretability and decision-making. ML systems in lung disease diagnosis benefit from regular validation based on real-world clinical results [10–12].
- This review analyzes ML approaches for diagnosing lung diseases. The main contribution of the research is:
- It investigates and addresses prominent lung diseases such as pneumonia, lung cancer, and COVID-19.
- It investigates and addresses the publicly accessible imaging modalities datasets for each prominent lung disease.
- It explores and addresses existing challenges and issues in diagnosing prominent lung diseases using ML and its associated novel solutions.
- It examines ML and its subfield approaches for identifying prominent lung diseases based on radiographic images and their significance.
- It qualitatively assesses ML approaches, emphasizing their efficiency in identifying, classifying, and forecasting prominent lung diseases while outlining essential considerations for enhancing the diagnosis.
- The particularity of the investigation is that it offers a conceptual context for the issues. Furthermore, the analysis emphasizes the techniques and primary methods used in the published findings.

The structure of the review is as follows: Section 2 explains the approach utilized to conduct this review and addresses the necessity of a study in light of recent research. Lung diseases and their classifications, following the most prevalent and well-researched trends, are described, as are the challenges in diagnosing lung diseases, in Section 3. In Section 4, the imaging modalities, both conventional and other types, are described. Section 5 discusses machine learning, its trends, prominent sub-fields, and the initial steps for applying machine learning to diagnosing pulmonary diseases. Section 6 presents the diagnosis of prominent lung diseases using ML and imaging and also comprises publicly accessible datasets for each one, along with extensive analysis and narratives. Section 7 provides observations and discussions. Section 8 concludes the review.

### Necessity

Multiple reviews/surveys/studies were examined, contrasted, and presented in Table 1 because of the tremendous relevance of correctly identifying prominent lung diseases using imaging modalities and ML.

**Table 1:** Comparative analysis of the review with recent researches

Ref.	Year	Type of Analysis	Focused Research	Lung Disease			Imaging Modality			Detailed Dataset	ML Methods					
				Type	S	M	X-ray	CT	O		ML	DL	CNN	TL	EM	O
[13]	2022	Review	Detection	Pneumonia	√	X	√	X	X	X	X	√	√	X	X	~
[14]	2022	Review	Diagnosis	Pneumonia	√	X	√	X	X	X	~	~	~	~	~	~
[15]	2022	Review	Detection and Classification	COVID-19	√	X	√	√	X	X	X	√	√	X	√	~
[16]	2022	Review	Diagnosis	COVID-19	√	X	√	√	√	X	√	√	√	~	~	~
[17]	2022	Survey	Detection and Diagnosis	COVID-19	√	X	√	√	X	~	X	√	√	~	~	~
[18]	2022	Survey	Detection	COVID-19	√	X	~	~	X	X	~	~	~	~	~	~
[19]	2022	Review	Classification	COVID-19	√	X	√	√	X	√	X	√	~	~	~	~
[20]	2022	Review	Prognostication and Detection	Lung Cancer	√	X	X	√	X	X	√	√	~	X	X	X
[21]	2022	Survey	Classification	Lung Cancer	√	X	X	√	X	~	X	X	√	X	X	X
[22]	2023	Review	Detection and Classification	Lung Cancer	√	X	√	√	X	X	X	√	√	X	X	~
This Review	2023	Review	Diagnosis, Detection, Classification, Prediction	Pneumonia COVID-19 Lung Cancer	√	√	√	√	√	√	√	√	√	√	√	√

*S* Single, *M* Multiple, *O* Others, *ML* Conventional ML Methods Applied, *DL* DL Methods Applied, *CNN* Convolutional Neural Network, *TL* Transfer Learning, *EM* Ensemble Method, *√* Discussed briefly, *X* Not Discussed, ~ Partially Discussed

As far as we know, previous research has yet to provide a combined comprehensive examination of identifying prominent lung diseases with ML and imaging modalities datasets. The methodology, procedures, and techniques of ML and imaging modalities are examined and brought to light in this research, which provides less time for understanding.

## Methodology

**Fig. 1:**
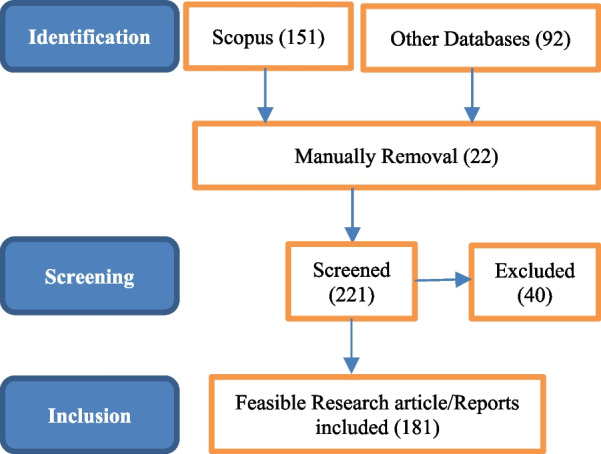
PRISMA flowchart

The Preferred Reporting Items for Systematic Reviews and Meta-Analyses (PRISMA) flowchart is depicted in Fig. 1, illustrating the approach taken. Establishing a suitable pre-existing research repository was essential for accessing scholarly research articles.

Scopus and Web of Science were preferred due to their prominence as widely used research databases for academic, peer-reviewed scientific papers. In addition, the well-known databases of academic studies that have been subjected to peer review, namely ScienceDirect [23], arXiv [24], IEEE Xplore [25], and MDPI [26], were also used for the search of articles. Only relevant published articles that are related to the issues are taken into consideration.

### Identification

Databases were searched using pertinent keywords to explore all feasible machine learning-assisted lung disease diagnosis publications. Applied keywords and combinations used to search procedures with primary considerations for review, such as lung diseases, imaging modalities, and ML, are presented in Table 2.

**Table 2:** Applied keywords for searching procedure

Major Considerations	Keywords
Lung Diseases	"Lung Disease" "Pneumonia" "Lung Cancer" "COVID-19" and "Coronavirus"
Imaging Modality	"X-Ray" "CT scan" "PET" and "MRI"
Machine Learning	"Machine Learning" "Deep Learning" "Convolutional Neural Network" "Transfer Learning" and "Ensemble Learning"

Studies were limited to articles written in English only. Only studies employing ML and its prominent subfields to diagnose lung diseases utilizing specific imaging modalities are included in this review. Studies that are deemed unimportant are excluded. 151 publications from the Scopus database and 92 articles, reports from Google Scholar, the website, and additional databases, including ScienceDirect, MDPI, and IEEE Xplore, were chosen at this round.

### Screening

The screening process ensured the selection of only relevant research. The review included only substantial titles and abstracts, not requiring a full-text assessment.

We manually eliminated duplicate titles, resulting in 22 remaining publications. Based on the screening, we selected 221 publications, excluding 40 due to irrelevance. All screened research publications pertained to an entitlement review.

### Inclusion

To conduct an entitlement review, we analyzed every research publication we examined. We evaluate each piece of research before considering it for assessment. At the end of this round, we found 181 viable studies/resources through manual investigation.

## Lung diseases

**Fig. 2:**
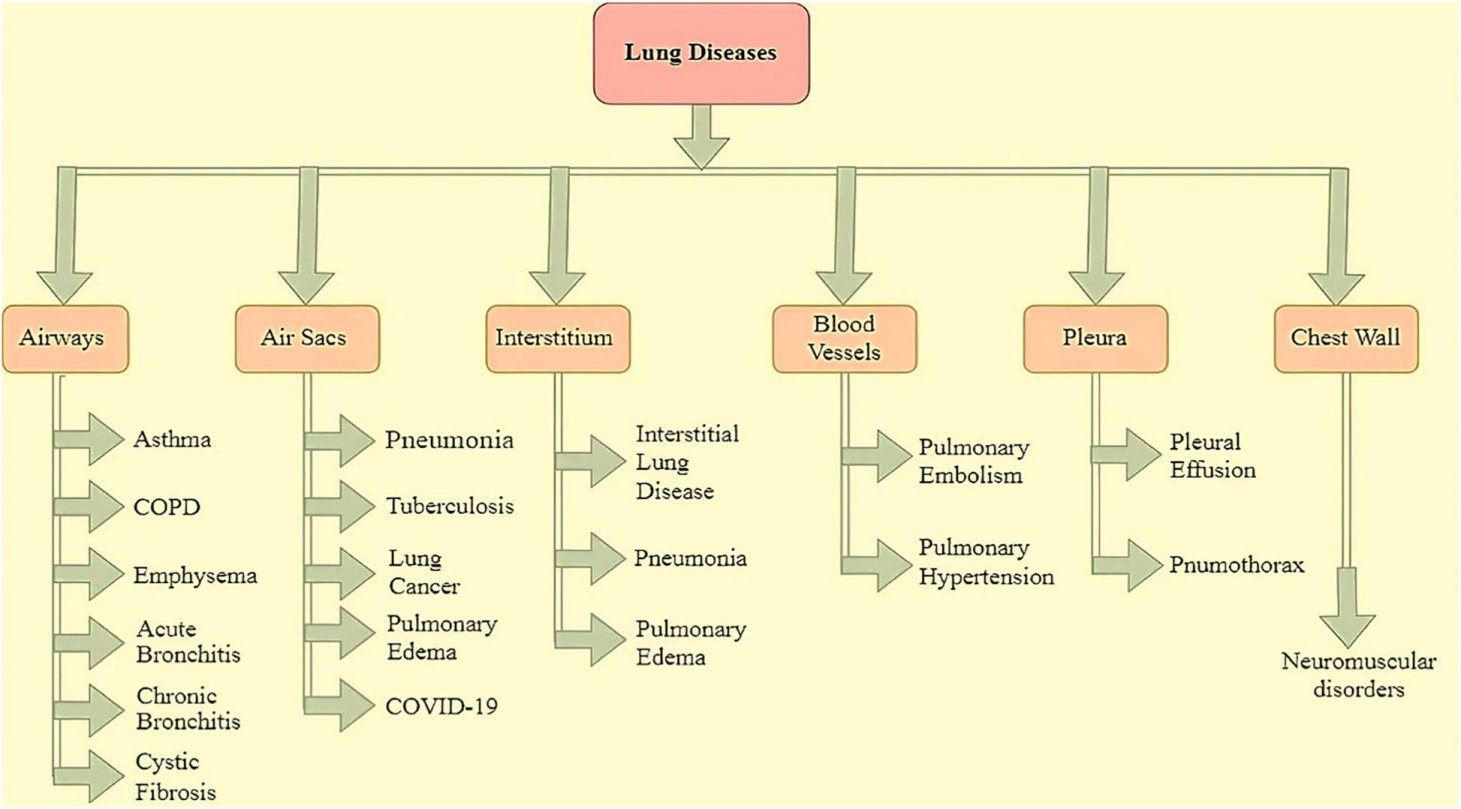
Types of lung diseases

Humans breathe by expanding and contracting their lungs to intake and expel oxygen, which is then circulated via deep lung arteries to generate energy for their bodies [27]. Lung diseases include a variety of ailments that influence lung function. These include obstructive, restrictive, and infectious diseases affecting lung structure and function. Lung diseases can be categorized as depicted in Fig. 2.

- *Airways-Related Lung Diseases*: The lung's windpipe, or trachea, is split into bronchi, branching into smaller tubes that extend throughout the lungs. Some conditions that might affect these airways include asthma, COPD, acute bronchitis, chronic bronchitis, emphysema, and cystic fibrosis.
- *Air Sacs-Related Lung Diseases*: The respiratory system comprises bronchioles and narrow passageways inside the lungs, terminating in clusters of alveoli, also called air sacs. These air sacs facilitate the formation of tissue in the lungs. Pneumonia, TB, emphysema, pulmonary edema, COVID-19, and lung cancer represent a selection of respiratory ailments affecting the lungs.
- *Interstitium-Related Lung Diseases*: The narrow, tiny membrane between the lung's alveoli is known as the interstitium. The interstitium is filled with tiny blood capillaries that facilitate the exchange of gases between alveoli and blood. A few lung conditions that impact the interstitium are interstitial lung disease (ILD), pneumonia, and pulmonary edema.
- *Blood-Vessels-Related Lung Diseases*: Low-oxygen blood is pumped into the right side of the heart through veins. It uses the pulmonary arteries to push blood into your lungs. These blood vessels can also acquire diseases. Pulmonary embolism and pulmonary hypertension are two lung disorders that impact blood vessels.
- *Pleura-Related Lung Diseases*: The pleura is a thin membrane surrounding the lungs and chest walls. A slight fluid coating with each inhalation permits the pulmonary pleura to slide down the wall. Pleural effusion and pneumothorax are pleural lung disorders.
- *Chest Wall-Related Lung Diseases*: The chest wall is essential to the respiratory process. The ribs are connected by muscles, enabling the lungs to expand. The diaphragm descends with each breath, which allows the lungs to enlarge due to the action. Neuromuscular problems, chubbiness, and hypo-ventilation disorder are all diseases that disrupt the chest wall [28]. After reviewing these categories of lung diseases, explaining each one in depth is difficult due to the numerous kinds. Our review focuses on humanity's most debilitating and catastrophic prominent lung diseases.

### Prominent lung diseases

According to the information mentioned before introducing the issue, the WHO recently produced research outlining the top 10 diseases responsible for the most fatalities worldwide. Lung illnesses, in all of their many facets, are accountable for the deaths of a disproportionately high number of individuals all over the globe. According to the WHO, lung infections like pneumonia are responsible for an estimated 16% of all deceases of kids below the age of 5 worldwide. It is also a top reason for hospitalization for kids below 5 in the United States [2]. According to the WHO, about 1.8 million fatalities a year may be attributed to lung cancer, putting it at the forefront of mortality due to cancer globally. It is responsible for more deaths than breast, prostate, and colorectal cancers combined. Most lung cancer cases are caused by tobacco use, with tobacco smoke being the primary risk factor for the disease [1]. COVID-19 is a well-known type of lung disease caused by the coronavirus. WHO is closely monitoring the ongoing outbreak of COVID-19. COVID-19 is a worldwide epidemic that has already infected almost every nation globally. The WHO reports showed that pneumonia, lung cancer, and COVID-19 are the three conditions that account for most fatalities. As long as COVID-19 persists, the world needs more investigations.

The most frequent lung conditions that may be identified using medical imaging are pneumonia, lung cancer, and COVID-19. This research's most prevalent lung diseases include pneumonia, lung cancer, and COVID-19. Each is described in depth below:

#### Pneumonia

Pneumonia is a leading cause of morbidity and mortality worldwide, surpassing other prevalent illnesses such as cancer, diabetes, HIV/AIDS, malaria, and several others. It is a severe lung condition with severe medical consequences and a high casualty rate in the short and long term. It is a common respiratory illness affecting the airways and alveoli. The development of pneumonia also depends on the patient's immune system's response to viruses. Patients who suffer from pneumonia exhibit pulmonary abnormalities [29]. There is a diverse array of microbes that are capable of causing pneumonia, such as bacteria, pulmonary pathogens, and fungi. Pneumonic microbial invaders are numerous and diversified. Pneumonia is caused by viruses such as coronavirus, rhinovirus, influenza, parainfluenza, metapneumovirus, and bacteria such as pneumococcus, mycoplasma, legionella, Enterobacteriaceae, Haemophilus, and mycobacteria [30].

#### Lung cancer

Lung cancer arises from the growth of cancerous cells within lung tissues, exhibiting uncontrolled proliferation that may spread to distant organs or lymph nodes. Lung tumors are divided into three groups from a histopathological perspective: small-cell lung cancer (SCLC), which also includes small-cell carcinoma; non-small-cell lung cancer (NSCLC); and other uncommon forms of tumors, which include sarcoma and lymphoma. Adenocarcinoma, squamous cell carcinoma, and large-cell lung cancer are the three subtypes of NSCLC [31]. Smoking is crucial in identifying lung cancer since it plays a critical function in the disease [32].

#### COVID-19

A specific contagious lung disease that spreads to people exponentially is COVID-19. COVID-19 symptoms include flu, cough, and shortness of breath. Less common symptoms include headache, decreased smell (hyposmia), decreased taste sensation (hypogeusia), throat infection, runny nose (rhinorrhea), muscle cramps, diarrhea, and vomiting. The main barriers comprise acute respiratory distress syndrome (ARDS), numerous organ failures, and death [29]. An RT-PCR (real-time reverse transcriptase polymerase chain reaction) test is the most modern and innovative way to detect COVID-19. COVID-19 might be classified.

##### Mild cases

An asymptomatic COVID-19 infection characterized by coughing, fever, and headache.

##### Moderate cases

Patients experience some shortness of breath as well as pulmonary issues such as hypoxia.

##### Complex cases

The patient is suffering from hypoxia as well as shock. This kind is to blame for the great majority of life-threatening incidents.

COVID-19 is putting the entire world in a horrific situation, bringing all life to a screeching halt worldwide and claiming millions of lives. As we have seen, when a pandemic occurs, there is a collapse in the healthcare system because we are unable to satisfy all the demands. The COVID-19 epidemic has significantly impacted medical microbiology labs. "Long COVID-19" or "post COVID-19 syndrome" refers to signs that may affect a person's health after recovering from the COVID-19 virus. These symptoms have been reported in many patients who have recovered from the COVID-19 virus [33].

#### Developmental analysis of prominent lung diseases over the internet

**Fig. 3:**
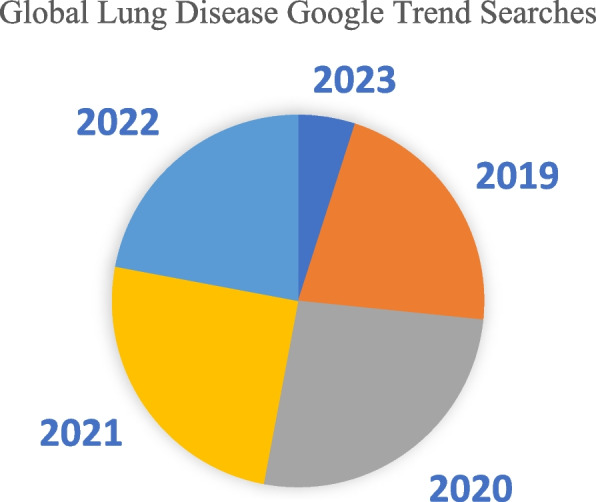
Worldwide lung disease searches on Google Trends

Google is the finest search engine for asking any question, and as almost every internet user utilizes it, it is frequently used to look for any query. So, it's helpful to know how people search for the most common lung disease on the internet. A well-liked and publicly available big data analytics tool called "Google Trends" has been extensively utilized to examine perceptions in several studies. Google Trends' tracking of internet search queries may offer some helpful insight. The searches for lung diseases from 2019 to 2023 were analyzed for this study (Fig. 3) [34].

**Fig. 4:**
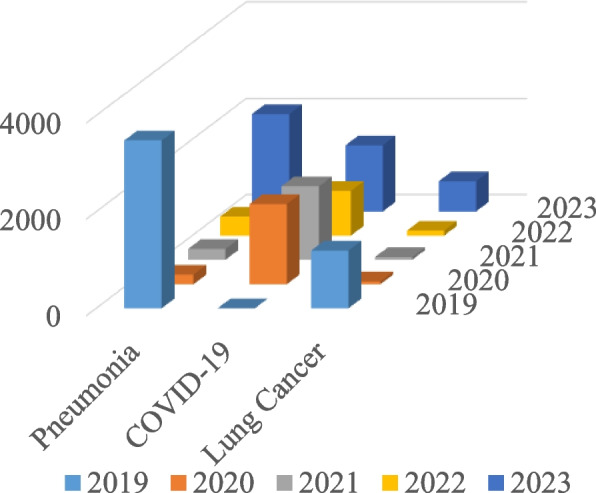
Worldwide Pneumonia, Lung Cancer and COVID-19 searches on Google Trends

Lung cancer and acute lower respiratory tract infections, which include pneumonia, asthma, COPD, and TB, are the five primary lung illnesses addressed at the International Respiratory Society Forum. Pneumonia is the top relative search term on Google Trends, according to Barbosa et al., who also noted that there has been an increase in COVID-19 pneumonia cases [35]. Since lung cancer is a fatal disease affecting individuals worldwide, it is commonly searched for online, mainly through research searches. Before 2020, there was a lower volume of COVID-19 searches, but during the pandemic, there has been an exponential increase in COVID-19 searches online. Search comparisons are necessary in the context of all lung diseases (Fig. 4).

The Y-axis in Fig. 4 displays the precise measurement numbering of Google Trends' searched queries, which illustrates the term's level of popularity [34].

#### Challenges and issues

Many lung disorders are avoidable but may go untreated due to a lack of diagnosis. Lung illness and other diseases, such as cardiovascular disease, sometimes coexist, yet combined diseases are usually misdiagnosed due to the significant overlap in symptoms [36]. When determining the presence of lung illnesses, there are several challenges to surmount. Some of them are as follows:

- *Selection of Efficient Imaging Modality*: Various imaging modalities, including X-ray, CT scan, SSMI, PET, and MRI, have been chosen based on clinical requirements [6–9]. Medical image analysis requires the selection of an efficient imaging modality for the detection [15, 19].
- *Scarcity of Useful Datasets***:** To handle and analyze medical images, an environment that supports access to medical data, data analysis, and processing is required [17]. Various imaging modalities datasets are available for public access [6–14, 23–26].
- *Effectiveness of Models Derived from ML*: The efficacy of models is crucial for identifying lung illnesses. If ML models are used, real-time diagnosis is essential. Thus, research on model training efficiency is necessary [30–33].
- *To Address Multiple Pulmonary Disorders Simultaneously*: It is expected that the trained ML model would be able to identify multiple lung diseases appropriately, such as COVID-19, pneumonia, and others [19–22].
- *Medical Experts' Opinions*: Although ML algorithms may be effective in classifying lung illnesses, medical expert evaluations and validations are required to confirm that the identification is correct [28–30].

## Imaging modalities

Diagnostic imaging is widely acknowledged to have a significant role in clinical evaluation. The processing of diagnostic imaging requires practitioners with extensive expertise. Healthcare practitioners may benefit from computer-assisted solutions due to diverse assessments of images, resulting in varying findings and a tedious process that may result in significant expenses and glitches. On the contrary, the manual diagnosis of lung disorders using radiographic scans often takes a substantial amount of time and is prone to error. The prompt and precise identification of lung disorders has a crucial role in enhancing the prognosis, thereby increasing the sufferer's likelihood of survival. The radiographic findings might be of assistance [37]. When a radiological image of a patient is produced, it is processed in many phases, including image annotation and segmentation. After storing the images in the databases, the radiologists annotated them after adding pertinent information to help the reader interpret them. Image segmentation is one of the most critical aspects of image processing. Images are divided around regions of interest (ROIs) to segment them [38].

With ethical concerns, the patient's clinical and radiological imaging must be processed while maintaining the subject's privacy. After receiving ethical consent, obtaining patient data, de-identifying it appropriately, and storing it securely is necessary. Pseudonymization is the technique of choice for de-identification since it replaces information that may be used to infer the identity of a subject with identifiers. When images are pseudonymized, you can't use this information to figure out who a patient is [39].

Labeled imaging data is commonly cited as a challenge for machine learning in the context of expanding medical imaging datasets. Therefore, various strategies that allow for learning with less or different sorts of monitoring are necessary [40]. The overview of each one is represented here for a better understanding.

### Conventional imaging modalities

#### X-ray

**Fig. 5:**
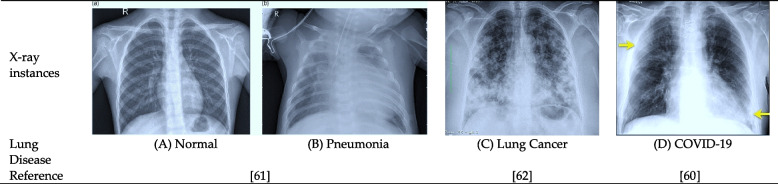
Instances of chest X-ray for prominent lung diseases

The chest X-ray (a CXR) is the diagnostic imaging method used most often in treating lung ailments. The availability, mobility, and cost-effectiveness of chest X-rays contribute to the initial evaluation of individuals exhibiting lung problems [3]. Since its earliest times, medical X-ray imaging has been captured on photographic films, which must be developed before being examined. Digital X-rays are used to solve this issue. The most popular medical X-ray diagnosis is a digital chest X-ray to diagnose lung disorders [41]. The vast majority of the analyzed studies used chest X-rays in their investigations. For instance, X-ray datasets were used for the diagnosis of pneumonia [42–55], lung cancer [44, 46, 47, 52, 56], and COVID-19 [47, 48, 53–55, 57–60]. Figure 5 depicts many chest X-ray illustrations of diverse lung diseases collected from publicly accessible datasets.

#### CT scan

**Fig. 6:**
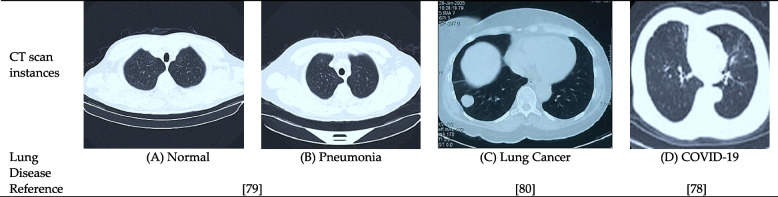
Instances of CT scans for prominent lung diseases

In patients with severe lung disorders, a chest CT is frequently recommended. CT imaging is more precise than CXR imaging and is employed when radiography reveals anything unclear [3]. By circling the X-ray tube around the chest, the CT merges several X-ray projections recorded from various angles to generate cross-sectional imaging of regions within the chest [6]. Chest CT scans were used in most of the studies reviewed for this study. For instance, the diagnosis of pneumonia [63], lung cancer [64–73], and COVID-19 [57, 59, 60, 74–78] relied on datasets that were acquired from CT scans. Figure 6 depicts many chest CT scan illustrations of diverse lung diseases collected from distinct publicly accessible datasets.

### Positron emission tomography

Nuclear imaging technology, such as PET, enables monitoring metabolic activities. It is done by injecting the patient with radiolabeled tracers and then figuring out where they went.

The most commonly used PET tracer is known as 18F-fluorodeoxyglucose (FDG). The disappearance of recognizable anatomical features is a defining characteristic of the PET imaging technique [6]. Lung disorders and nodules may be effectively evaluated with PET. It has an outstanding capacity for detecting metastases [81].

**Fig. 7:**
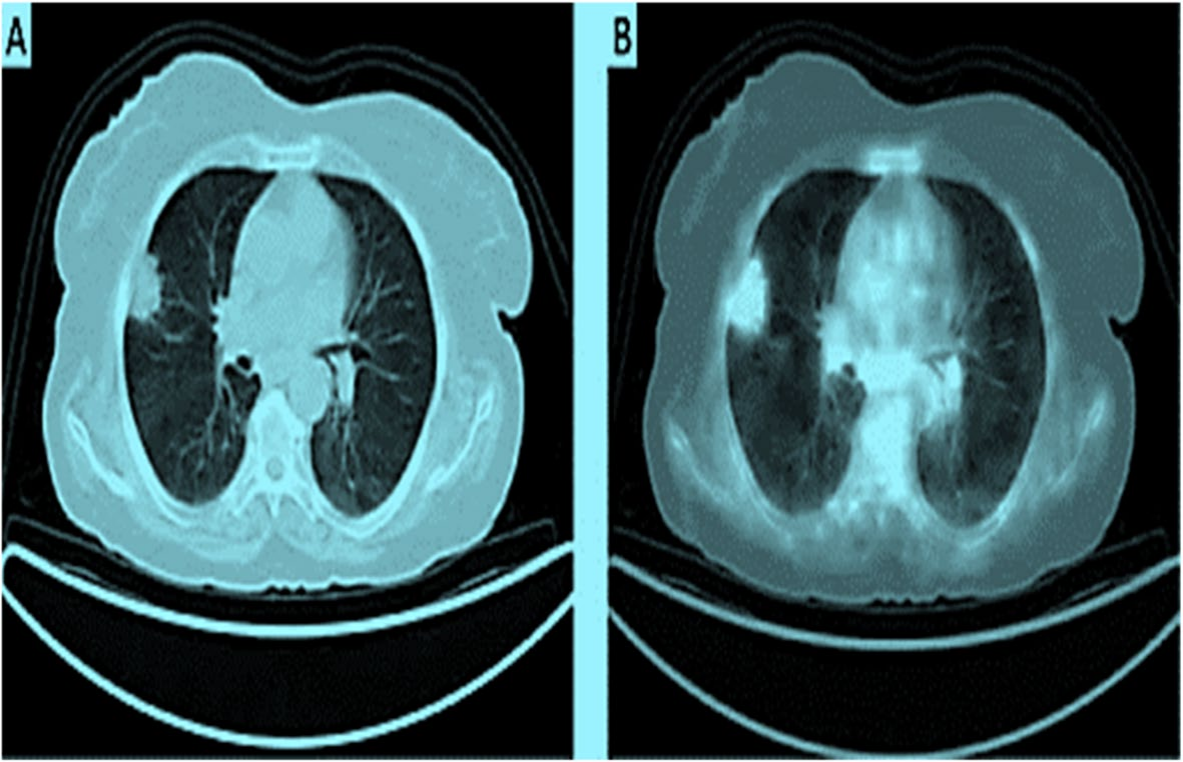
**A** A CT scan reveals a nodule in the anterior portion of the right lung's upper pole. **B** On 18F-FDG PET/CT, the lung nodule exhibited enhanced focused uptake, indicating a malignancy [82]

Figure 7 displays a chest CT scan of a lung nodule compared to a PET image, which provides a more improved view. The image was obtained from the Openi website, which provides access to publicly available images.

### Magnetic resonance imaging

Comparing MRI to other radiography modalities like CT, and Comparing MRI to other radiography modalities like CT and PET, it becomes evident that MRI has little clinical use for patients with lung illnesses. MRI generates images of the region that has been chosen and exhibits them in the form of narrow slices that comprise the entire volume of the area. It did work because nuclei absorb radio frequencies when powerful magnetic fields are present. MRI employs a magnetic field and radio waves to obtain numerous images of the lungs' region from various angles. Combining these images may generate crisp and accurate portrayals of areas [81]. Lung MRI is an excellent technique for doing sequential follow-ups [7]. MRI procedures like three-dimensional gradient sequences and acceleration techniques, among others, have increased MRI's minor lesion detection capabilities [83]. Also, research has shown that MRI might be a better way to screen for lung cancer than low-dose CT [84].

**Fig. 8:**
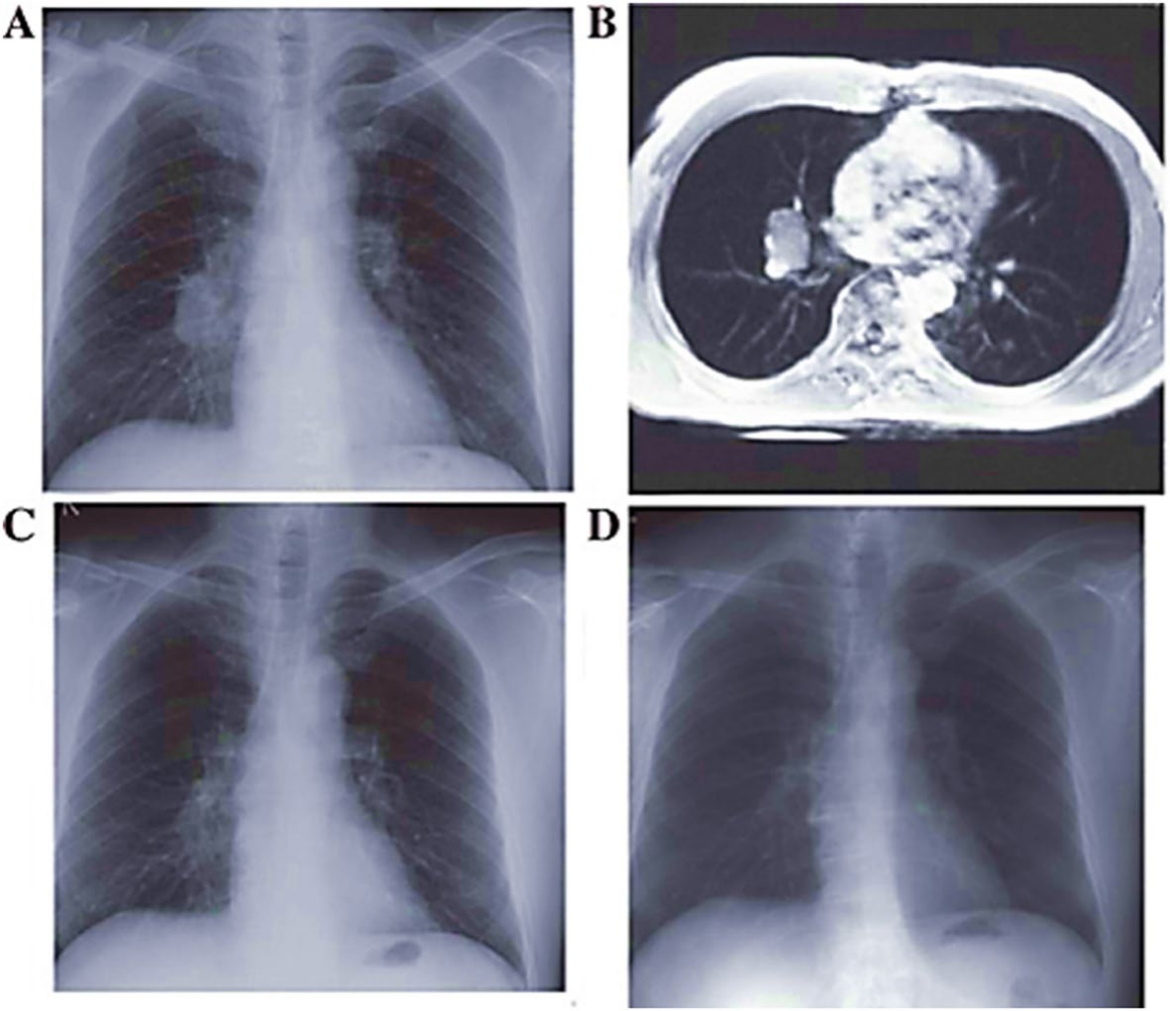
Chest X-rays and MRI (**A**) A lesion in the right hilus pulmonis with a clear edge is seen on a chest X-ray. **B** An MRI shows a nodule in the right hilum. **C** A chest X-ray shows no mass but a tangled network of blood vessels (**D**) A normal chest X-ray [85]

Figure 8 displays the chest radiograph of a lung nodule compared to an MRI image. The image was obtained from the Openi website, which provides access to publicly available images.

#### Sputum smear microscopy images

**Fig. 9:**
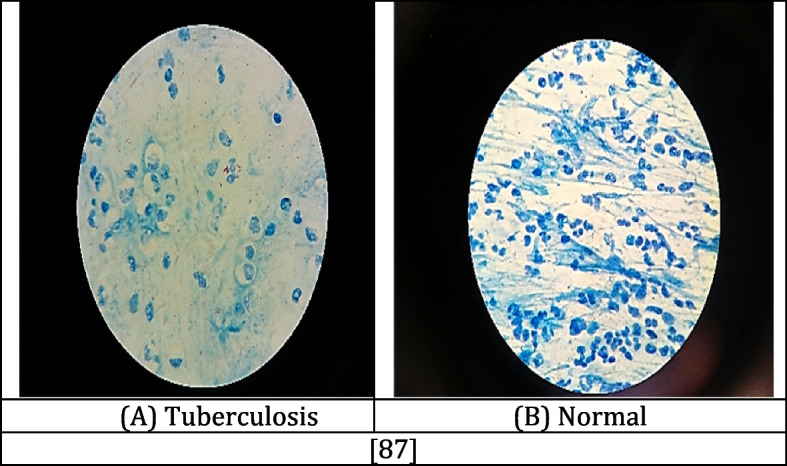
Instances of SSMI for tuberculosis

A viscous fluid called sputum is produced in the lungs and air passages, which is a crucial factor in the progression of certain lung disorders. Sputum smear microscopy has generally been considered the most effective approach for diagnosing lung diseases like TB. Specimens of sputum expectorated by patients with symptoms are placed chemically onto plain glass microscope slides [8]. Then, they are analyzed by laboratory procedures that identify acid-fast bacteria (AFB), like Mycobacterium TB cells [86]. The images obtained from a sputum smear test are often obtained via fluorescence microscopy or conventional microscopy. SSMI images were captured using a digital microscope and a digital camera. The captured images have a specific size and resolution depending on the magnification. The "pixel pitch," which refers to the physical size of each image pixel, is measured in micrometers [87]. Figure 9 displays SSMI images. The image was obtained from the open-access dataset [88], which provides access to publicly available images.

### Molecular imaging

Molecular imaging methods not previously used are also being studied to learn more about lung diseases. It is a specific type of imaging technique that combines the two fields of molecular biology and medical imaging. Recent research has been conducted on several methods of molecular imaging that have the potential to differentiate between the cellular and molecular components of respiratory illnesses. Alternative imaging techniques like single photon emission computed tomography (SPECT) can offer pertinent data at the molecular level because of their remarkable sensitivity and resolution. When it comes to the exactness of a lung diagnosis, the stage of the disease, or monitoring after treatment, molecular imaging may be a great addition to traditional imaging methods [9].

### At-bedside imaging modalities

Evolving methods can assess, monitor, or measure lung disorders at the bedside. Bedside methods, including lung ultrasonography (LUS) and electrical impedance tomography (EIT), are gaining prominence alongside conventional imaging modalities. Since they do not require ionizing radiation and are very uncomplicated, these approaches are being intensively explored as an addition to traditional procedures and, in the case of specific lung problems, as a substitute for them [89].

Following is an overview of the numerous imaging modalities. It has become clear that each characteristic sets it apart from the others. Every imaging modality collects its own specific set of images, enabling radiologists to identify a variety of lung illnesses more accurately.

## Machine learning

ML is a crucial component that can add resiliency to the medical decision-assistance systems. To better understand ML-based lung disease diagnosis, we provide a new analysis viewpoint on the different machine-learning strategies. The strategies for ML include supervised, unsupervised, and semi-supervised learning. Each method has benefits and drawbacks, and the selection of ML methodology hinges on the nature of the need [90] and the virtues and limitations listed in Table 3.

**Table 3:** Virtues and limitations of the various ML strategies

ML strategy	Virtues	Limitation	Preferred Diagnoses	Reference
Supervised Learning	- Assists in resolving issues with training data - Provides results with good performance measures - Task driven approach - Classification and Regression	- Training data must be labeled - Input data must be of good quality with adequate data	Pneumonia	[91]
Unsupervised Learning	- It works best with unprocessed or raw data - Data driven approach - Clustering and Dimensionality Reduction	- Does not employ a feedback mechanism to evaluate the standard results	Lung Cancer	[92]
Semi-supervised Learning	- Data with labels and without labels can both be used - Classification and Clustering	- Unable to handle unobserved data	COVID-19	[11]

In supervised learning, the ML model has the input–output pair along with the labeled data [91], whereas in unsupervised, the model only has the input data without any labeled data. Unsupervised learning examines standard results without feedback mechanisms. This strategy extracts features to cluster input data into groups to train the model. The technique finds an unusual pattern in the input data [93]. On the other hand, semi-supervised learning can work with both labeled and unlabeled data [11]. This strategy can operate on massive amounts of data due to the applicability of labeled and unlabeled data, even though labeled data are limited.

The general assumption is that performance measures acquired from labeled data will perform better than those obtained from unlabeled data. This assumption, however, is only sometimes accurate since the researchers demonstrated that unlabeled data may also provide remarkable performance measures [94].

### Machine learning developmental analysis on the internet

**Fig. 10:**
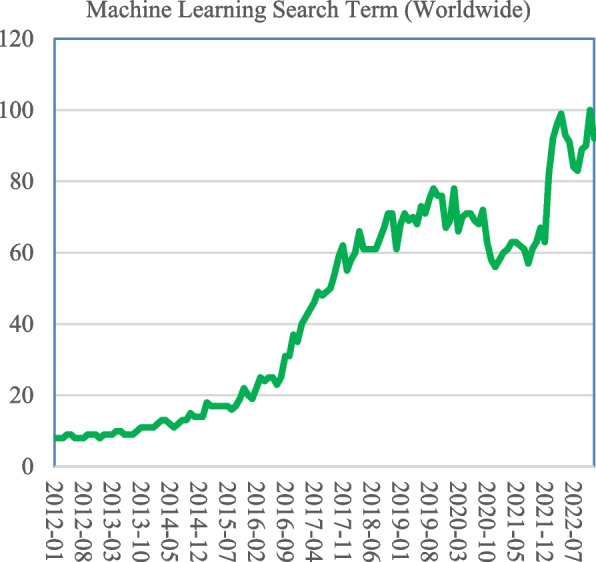
Machine learning searched the internet internationally

Since the turn of the decade, people worldwide have searched the internet using the term "machine learning." The Y-axis in Fig. 10 displays the precise measurement numbering of Google Trends' searched queries from 2012 to 2023, which illustrates the term's level of popularity [95]. Such statistics motivate the research of machine learning in the context of the study of the detection of lung diseases. The popularity of ML is seeing meteoric growth.

### Introductory steps for employing machine learning to diagnose lung diseases

**Fig. 11:**
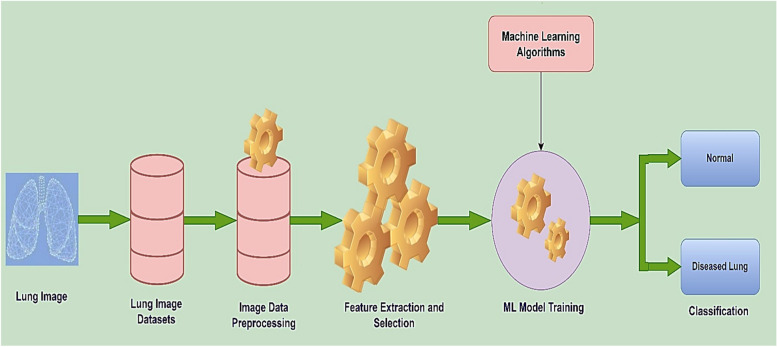
Lung disease diagnostic pathway with ML

ML has the potential to diagnose and prognosticate lung illnesses. To make a diagnosis using imaging modalities, ML executes a series of actions, including acquiring an image dataset, preprocessing the image data contained within the dataset, performing feature extraction and selection, training an ML model using specific ML algorithms, and evaluating performance metrics and classification [96]. The lung disease diagnostic process using ML is shown in Fig. 11.

**Fig. 12:**
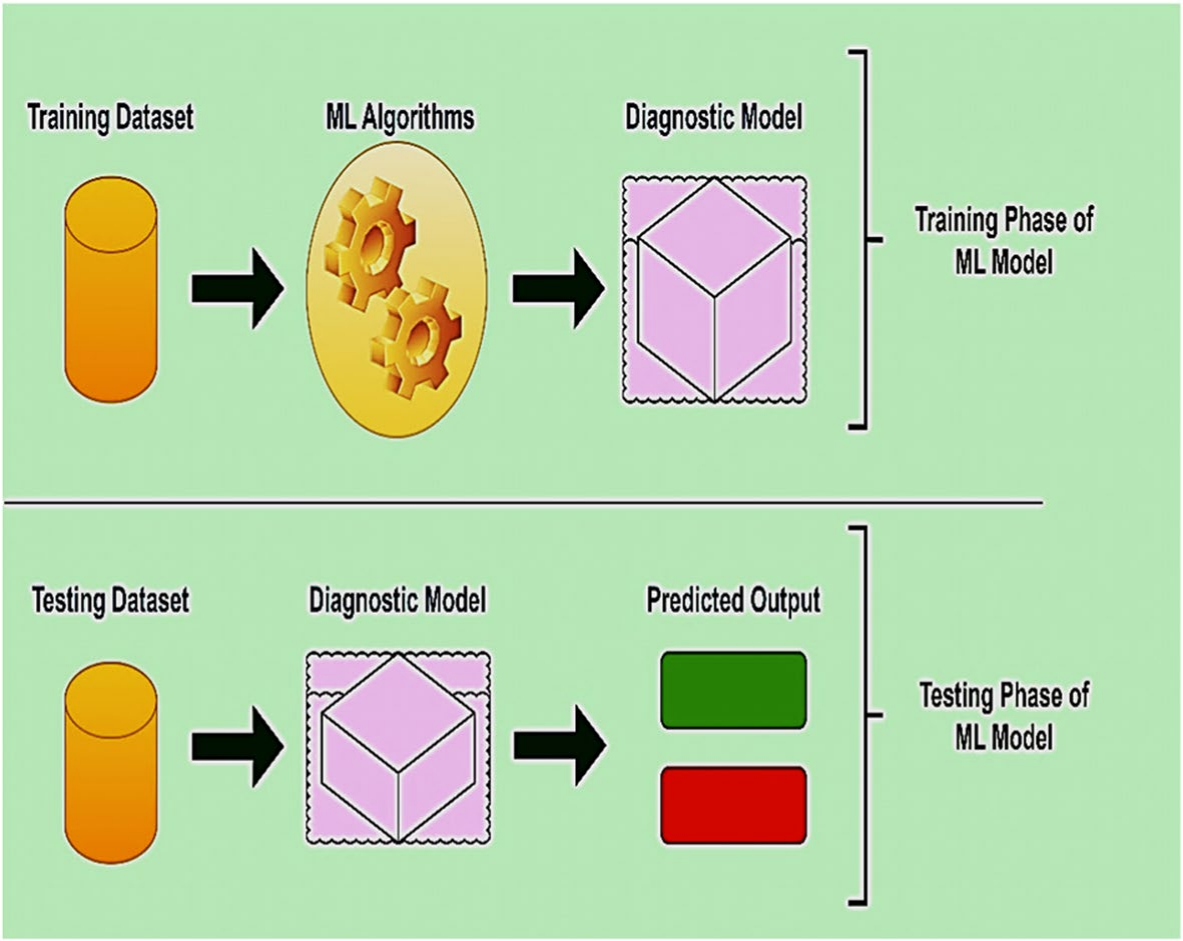
ML diagnostic model from the viewpoint of the training and testing phases

The above-described introductory steps for employing ML to diagnose lung diseases act as the training phase of the ML model, which develops an ML diagnostic model. However, this ML diagnostic model must be validated using new or test data that the model has never seen before. Machine learning advances the lung disease diagnostic pathway. The fundamental framework of an ML-based diagnostic model is shown in Fig. 12, in which the model is trained using a training dataset and evaluated using new test data.

Many imaging modalities make it possible to record data about a patient's lungs from various angles and viewpoints, which may then be annotated and stored for later use [97].

Collecting these images produces an image dataset that can be preprocessed and employed as an input for the ML to operate on [98]. The necessary features must be retrieved and selected manually or automatically from the preprocessed picture dataset to train the model using any particular machine learning algorithm [99]. It is possible to do prediction or classification using a trained model [100]. It is a conventional approach to ML for diagnosing lung diseases using imaging modalities.

### Publicly accessible datasets

In the modern world, data is far too important. According to one of the studies of digital health records, it was discovered that around 25 million images were subject to cyberattacks [101]. Assume that the European Union (EU) has enacted special regulations for data protection. The General Data Protection Regulation (GDPR) is a form of legislation that updates and unifies data privacy rules across the EU and its associated businesses. Due to GDPR in the EU, hospitals and other healthcare organizations cannot share data [102]. Data sharing for research and other specific purposes is limited, encouraging private or commercial data use.

In contrast to private or commercially supplied datasets, which are not openly accessible to the research community, publicly available datasets are preferable since they are accessible to all researchers and can be used for their studies. The imaging modality appropriate for the particular lung disease must be ascertained first. Certain lung disorders are diagnosed using imaging techniques such as X-rays, CT scans, SSMI, PET scans, MRIs, and others as specified earlier [103]. A dataset must be compiled based on specific images, which may be either public or private. A researcher may collect or create private datasets depending on the research demands. However, a researcher or organization may also provide publicly available datasets if they wish to make their findings public. Researchers developing ML models must access such a vast dataset of these modalities [104].

### Preprocessing

Preprocessing the dataset is essential after choosing a particular image dataset. An image dataset's description, visualization, and other attributes can all be used for analysis. It is necessary for the exploration to collect relevant image data for the ML model of lung illness. The ML model heavily depends on image quality for training. Dealing with real-world imaging data requires a more in-depth examination of the data collection process. Several images may need clarification, including incomplete annotations, anomalies, and nonsensical image data within the obtained image dataset. It is challenging to clean and preprocess image data received from databases correctly. Hence, adapting or implementing appropriate preprocessing techniques is necessary [105].

Image enhancement and optimization may be done using ML-based image processing [106]. Approaches to image processing based on AI can lessen the amount of time needed for the process while improving image processing techniques. When preprocessing an image, it can be transformed into a grayscale and cleaned up with Gaussian blur, median filters, morphological smoothing, and numerous other methods [107]. Contrast Limited Adaptive Histogram Equalization (CLAHE) is one of the famous techniques that can be employed to improve the image's contrast [108]. Image processing techniques like lung segmentation, which necessitates the exclusion of bone, might be used to locate the region of interest, after which lung disease detection could be carried out in the region of interest [109].

### Feature extraction and relevant feature selection

Certain extracted features may be valuable, while others will not. That ultimately leads to the identification of relevant components. ML algorithms or Classifiers process these features selected for analysis. The feature engineering method consists of two segments: the first aims to extract parts from an existing image dataset, and the second involves picking features among the extracted ones. Methods like Gabor, Zernike, Haralick, and Tamura were used to extract features [110]. Features may be selected using techniques like the gray level co-occurrence matrices (GLCM), local binary pattern (LBP), and CNN. The bio-inspired algorithms such as the improvised crow search algorithm (ICSA), the improvised grey wolf algorithm (IGWA), and the improvised cuttlefish algorithm (ICFA) are all examples of feature selection algorithms that can be used to narrow down a large number of acquired features to only the most desirable ones. Genetic algorithms can also choose diagnostic imaging features [111].

### Training of the machine learning model

ML model training is the primary process of the ML pathway, providing an effective model for assessment, verification, and distribution. The ML model has been trained with the help of the relevant available data and can be used to analyze newly collected data and provide predictions utilizing the model [10].

Following the partitioning of the image database, one segment is expected to be set aside for the training phase of the ML model and another for the testing phase. The test data consists of novel data that will be employed in the future to assess the effectiveness of the ML model. Knowing the significance of training in ML will enable the system to collect the appropriate volume and quality of training data for the model. Once the system knows how it affects model prediction and why it's essential, it can choose the optimal algorithm based on the availability and suitability of the training data set [112].

### Machine learning and its algorithms

The ML algorithm enables the ML model to perceive the input data in a particular manner. The training process is the sole method that interoperates with ML algorithms so that ML models can extract meaningful information from learning data. It might take time to find an algorithm that works well and is set up to meet the needs of the intended use in a particular domain. Distinct learning algorithms have different objectives, and their results may vary based on data features. So, it's essential to know about machine learning algorithms and how they work in the real world, such as in medicine and other fields [113].

There are many different kinds of ML algorithms. Some are based on regression, decision trees, the Bayesian method, the kernel method, the clustering method, the ensemble method, and artificial neural networks (ANNs) [105].

- Regression is a common technique for reducing model-based uncertainty by iteratively adjusting the model in response to the errors it produces. Some types are linear, logistic, stepwise, and multivariate adaptive regression splines (MARS).
- To predict the target variable based on the input variables, an algorithm in the form of a decision tree is utilized. Some examples are random forest, classification and regression tree (CART).
- Those algorithms that are based on the Bayesian technique are the ones that use the Bayes theorem and make it easier to use subjective probability in model development. The significant algorithms used for classification and regression problems are Nave Bayes and Bayesian Belief Network.
- Pattern analysis is the basis of the kernel approach, which incorporates a wide range of mapping methods. Support vector machines (SVM) and linear discriminant analysis (LDA) are essential kernel approaches in ML modeling.
- By grouping data points according to their similarities, clustering is the most widely used unsupervised learning approach. K-Means, partitioning-based, hierarchical, and density-based clustering are just a few examples of clustering techniques that may be classified in various ways.
- Ensemble methods are strategies that work on several models and unite them to obtain more accurate outcomes. Compared to relying on a single model, the results of ensemble techniques are often more reliable. Bagging, boosting, AdaBoost, gradient boosting machine, and random forest are prominent ensemble techniques.
- Simulations on a computer based on biological principles are used for various purposes, including clustering and classification. There are many ways to use ANN, such as the perceptron, the Hopfield network, and backpropagation.

### Performance metrics

Building an ML model is not sufficient; the evaluation of the build model is to ensure its reliability and forecasting. Performance metrics are a set of statistics used to assess an ML model's overall efficacy and efficiency. These metrics can be quantitative or qualitative, and they can evaluate many aspects of performance. Typically, they oversee improvement and progression over time [114]. The majority of researchers, while conducting their studies, make use of a range of vital metrics, some of which are as follows:

- Accuracy: The accuracy of an ML model is measured as the proportion of correctly classified samples to the total samples. It is the most common metric used to measure the performance of an ML model. It can be expressed as (Eq. 1):
  $$\mathrm{Accuracy\,}=(correctly\,classified\,samples)\,/\,(Total samples)$$

The correctly classified samples can be expressed as follows:

$$\mathrm{correctly\,classified\,samples\,}=\,True\,Positive\,\left(TP\right)+\,True\,Negative\,(TN)$$

The total samples can be expressed as follows:

$$\mathrm{Total\,sample\,}=\,TP\,+\,False\,Positive\,(FP)\,+\,TN\,+\,False\,Negative\,(FN)$$

- Sensitivity: This metric measures how many relevant samples an ML model can identify by calculating the proportion of true positives to all actual positives and presented through Eq. 2. It is often called the "true positive rate" and the "recall."
- Precision: This metric measures how accurate a model's predictions are by calculating the ratio of true positives to all positive predictions made by the model. It is often referred to as "positive predictive value" and is presented through Eq. 3.
- Specificity: It measures how well a model can correctly identify negative samples. It is the ratio of true negatives that are correctly identified and presented through Eq. 4. An ML model with high specificity may have a low false-positive rate, meaning it will rarely incorrectly classify negative examples as positive.
- F1 Score: This amalgamation of precision and recall scores provides an overall score for model evaluation. The F1 score is presented in Eq. 5.
- AUC: AUC stands for Area Under the Receiver Operating Characteristic Curve. For varied thresholds, AUC graphs the actual positive rate versus the false positive rate, which is used to evaluate a model's ability. The AUC represents the degree of discrimination between classes [115]. Some of the performance metrics are presented in Table 4.

### Classification of lung diseases

Classification identifies, comprehends, and groups objects and concepts into predetermined categories. The act of classifying something is pattern recognition. Classification is a specific type that predicts a class label for a given sample Table 4.

**Table 4:** Performance metrics

Metric	Equation	
Accuracy	$Accuracy = \frac{\left(TP+TN\right)}{\left(TP+FP+TN+FN\right)}$	(1)
Sensitivity	$Sensitivity= \frac{TP}{TP+FN}$	(2)
Precision	$Precision = \frac{TP}{TP+FP}$	(3)
Specificity	$Specificity= \frac{TN}{TN+FP}$	(4)
F1 Score	$F1 Score = 2*\left(\frac{Precision*Recall}{Precision+Recall}\right)$	(5)

It transforms a function from input to output variables as a target, label, or class. "binary classification" describes classification tasks with just two possible class labels. Classification problems with more than two categories are called "multiclass classification." Some of the algorithms developed for binary classification can also address multiclass concerns [105].

## ML sub-fields

Numerous prominent sub-fields of ML may be utilized to diagnose lung diseases. Deep learning (DL), CNN, ensemble techniques, transfer learning, and many other notable ML subfields may be used to diagnose lung conditions. Many more subfields of ML can also be employed. The focus here is on elaborating on a few particularly notable sub-fields.

### Deep learning

A popular and rapidly developing area of ML is DL. Learning A popular and rapidly developing area of ML is DL. Learning from massive datasets is the focus of DL, a subfield of ML that employs neural networks. DL enables the creation of diagnostic models by performing all the processing steps typically associated with the construction of standard ML models, such as feature extraction and selection, in an automated manner. The word "deep" signifies that many hidden layers comprise the neural network. There is a particular set of neurons in the processing layers of neural networks for deep learning. The first layer in a network is known as the input layer, the final layer is known as the output layer, and the layers in between are known as the hidden layers [116]. DL has been influential in diagnostic imaging for feature engineering and image classification [117] and can resolve data-related problems with minimal supervision. It has consequently prompted researchers to research DL approaches at deeper levels. DL algorithms do exceptionally well compared to conventional differential diagnosis screening processes that rely solely on radiologists [118].

Consequently, DL offers novel models for classification tasks and medical image diagnostics [119], which achieve excellent results. In particular, DL approaches are anticipated to aid physicians in the examination and diagnosis processes [120]. DL leverages ANN to examine raw data directly. Multilayer perceptrons (MLP) also comprise the most prevalent deep learning algorithms.

Three primary groups of DL approaches are supervised, unsupervised, and semi-supervised. Several supervised learning approaches include CNN, deep neural networks (DNN), and recurrent neural networks (RNN). DL excelled in non-linear dimensionality reduction and clustering problems in unsupervised learning. It comprises limited Boltzmann machines, auto-encoders, and generative adversarial networks (GANs). Semi-supervised deep understanding also includes GAN. In addition, RNNs, which contain GRUs and LSTM techniques, could be applied to all ML strategies, such as supervised and unsupervised learning [121].

**Fig. 13:**
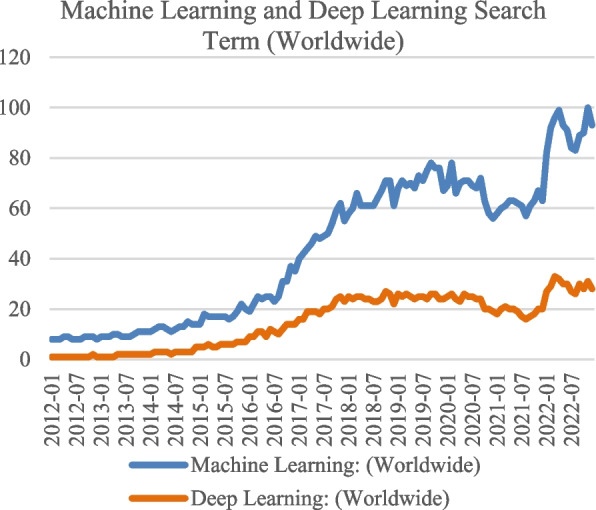
Machine learning and deep learning searched the internet internationally

A decade-long comparison of the search volumes for "Machine Learning" vs. "Deep Learning". Figure 13 depicts the Google Trends queries performed between 2012, and 2023. Results indicate that ML searches predominate over DL searches due to their use as an umbrella term [122].

### Convolutional neural network

**Fig. 14:**
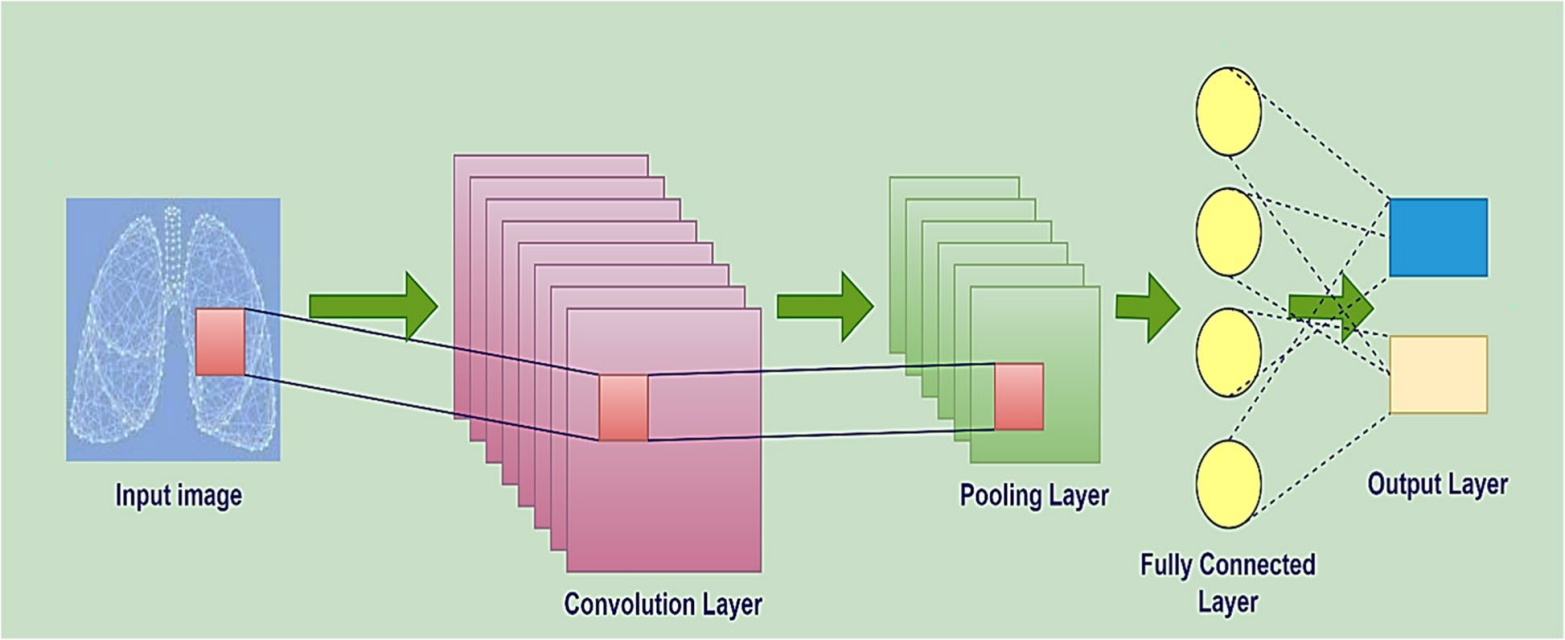
Basic architecture of CNN

CNNs were implemented in several domains, including computer vision and medical imaging. In particular, CNNs have been effective at producing outputs in previously unattainable settings [123]. It is the case since CNNs can detect and learn crucial traits that radiologists cannot readily observe with visual inspection [124]. CNN's primary advantage over its earlier works is that it intelligently recognizes pertinent features. There are many advantages to utilizing CNNs, including the feature of weight sharing, simultaneously learning both the feature extraction and the classification, and the capability to create large-scale networks [121]. The basic architecture of CNN is represented in Fig. 14.

### Convolutional layer

The convolutional layer comprises a procedure that involves repeating a specific filter over the whole image. The incoming image (i) of every layer in the model of CNN is presented in three dimensions: height, width, and depth, represented as *a* × *a* × *b* in the dimensional form, in which the height (*a*) is the same as the width (*a*). A different name for depth (*b*) is the channel number. 

Filters may have a variety of sizes, including 3x3, 5x5, 11x11, etc. Filters convolutionally transform the preceding layer's inputs into the corresponding layer's output. A feature map is produced as a result of this convolution procedure.

*k* is the number of kernels, also known as filters, contained within every convolutional layer with the same dimensional form as the input image, represented as *c* × *c* × *d*, with the following conditions: *c* < *a*, and *b* <  = *d*. A dot product is computed between the inputs of the convolution layer and the weights of that layer. To generate k feature maps (*h*^*k*^) as presented in Eq. 6, input is convolved with these kernels, which all have the same bias (*b*^*k*^) and weight (*w*^*k*^) [121, 125].

6 $${h}^{k}=f\left({w}^{k}*i+{b}^{k}\right)$$

#### Activation functions

All activation functions in neural networks that deal with non-linearity map input to output. The input value is calculated by weighting the neuron input and adjusting for bias. CNN and other types of deep neural networks often use the Relu, Leaky Relu, and Noisy Relu, as well as the Sigmoid and Tanh activation functions. An activation function that may prevent vanishing gradients is the rectified linear unit (ReLU). This interpretation focuses on the argument's positive axes [121]. Some of the prominent activation functions that are widely used are presented in Table 5.

**Table 5:** Prominent activation function

Ref	Activation Function	Output Range	Equation	
[121]	Relu	0 to ( +) Values	${f\left(x\right)}_{Relu}\text{= Max(0, x)}$	(7)
[126]	Sigmoid	0 to 1	$\updelta \left({\text{x}}\right)=\frac{1}{1+{{\text{e}}}^{-{\text{x}}}}$	(8)
[127]	Tanh	(-) to ( +) Values	$Tanh(x)=\frac{\left({e}^{x}-{e}^{-x}\right)}{\left({e}^{x}+{e}^{-x}\right)}$	(9)

#### Pooling layer

A down-sampling operation must be done on each feature map in a pooling or subsampling layer. A pooling layer is characterized by a formation that preserves the image features while simultaneously reducing the image size. Additionally, it stores image information. This subsequent step is to use a pooling function, such as maximum, global, or average, with a kernel size or pool size that has already been set for each of the feature maps [125].

#### Optimizers

Updating the weights in the CNN architecture requires employing optimization algorithms at each level until it is possible to get the maximum learning. The updating procedure is carried out by each approach using its unique algorithm. Some of the best-known optimizers are called Gradient Descent, Stochastic Gradient Descent, and Adam [125].

#### Fully connected layer

It is a layer in which every precomputed input node is coupled to every output node. It is a layer utilized to make predictions at the network's end. This layer connects each neuron of the preceding layer to each neuron of the current layer. The previous layer's output is flattened and delivered to a fully connected layer that linearly modifies the data before sending it to a nonlinear activation function [128].

#### CNN architectures

Various CNN architectures carry out classification tasks, including ResNet, VGG Net, Inception, Xception, DenseNet, EfficientNet, MobilenetV2, and many more. On the other hand, segmentation tasks are carried out by U-Net, V-Net, FCN, SegNet, DRUNET, and many different architectures [129]. With the aid of CNN, the number of parameters can be significantly reduced, overfitting can be prevented, and the information gleaned from an image may be preserved.

### Ensemble learning

Ensemble learning aims to improve general performance by integrating different models into a single one. It was initially proposed for classification tasks. The benefits of both deep learning and ensemble learning are combined in deep ensemble learning models to provide a model with enhanced performance [130]. An ensemble of learned models may be created by taking the training data, deriving many training sets from it, learning a model from each, and then combining them. The bagging, boosting, and stacking methods are all well-known ensemble learning methods. The result of combining model outputs is a single prediction. A weighted vote facilitates classification, whereas a weighted average reduces numerical prediction. This approach is used by bagging and boosting; however, their respective models are generated uniquely [131]. Stacking enables the combination of fundamental learning algorithms. Diversified foundation models allow the stacked ensemble to learn from various perspectives, producing heterogeneous features. The super learner approach is called "layered ensemble learning" [132].

### Transfer learning

ML approaches only function when testing and training data are from the same feature space and dispersion. Statistical models must be reconstructed with fresh training data when the dispersion changes. In many instances, based on the real world, retrieving data for training and recreating models is either impractical or too expensive. It would be helpful to reduce training data collection work. In certain circumstances, transfer learning across task domains is advantageous. Whenever there is inadequate standard training data for a given job, one solution is to use transfer learning methods to bring the knowledge acquired from previously experienced tasks to the target job [133]. Inductive [134] and transductive kinds of transfer learning are preferred for classification or regression studies. On the other hand, unsupervised types of transfer learning are selected when it comes to tasks involving clustering and dimensionality reduction [135]. Transfer learning made the DL model even more accurate by fine-tuning it with more training data and adjusting the parameters.

## Detection of prominent lung diseases using machine learning and imaging

The backbone of ML models is input data, which comes in the form of datasets and ML diagnostic methods. Therefore, at first, the primary emphasis of this review was on the datasets that were given for the prominent lung diseases, and the subsequent section discussed the ML approach for the diagnosis in more depth.

### Publicly accessible datasets

#### Pneumonia

To initially address the issue of accessing image data, public datasets are preferred and represented since virtually everyone can access them, which makes them ideal for conducting research. This section summarizes the publicly available pneumonia datasets used in the reviewed study to provide readers with relevant data for the datasets on pneumonia. The datasets for the diagnosis of pneumonia that are publicly available are listed in Table 6.

**Table 6:** Available pneumonic datasets

Dataset Name	Pneumonia Types	Modality	Number of Images	Reference
Large Dataset of Labeled Optical Coherence Tomography and Chest X-Ray Images (LDOCTCXR)	Viral Pneumonia Bacterial Pneumonia	X-Ray	Total – 5,232 3,883—Pneumonia 1,349 – Normal	[42]
Radiological Society of North America (RSNA)	Pneumonia Normal	X-Ray	Total – 5,528	[43]
NIH Chest X-rays (ChestX-ray8)	Pneumonia And 7 others	X-Ray	Total – 1,08,948 1,062—Pneumonia 84,312 – No Findings	[44]
NIH Chest X-rays (ChectX-ray14)	Pneumonia And 13 others	X-Ray	Total – 1,12,120 1,353—Pneumonia 60,412 – No Findings	[46]
Chest computed tomography in COVID-19 pneumonia	COVID-19 Pneumonia	CT scan	105 – Positive	[63]
Curated Dataset for COVID-19 Posterior-Anterior Chest Radiography Images (X-Rays)	Bacterial Pneumonia Viral Pneumonia COVID-19 Normal	X-Ray	Total – 9,208 3,001- Bacterial Pneumonia 1,656—Viral Pneumonia 1,281—COVID-19 3,270—Normal	[47]
Balanced Augmented Covid CXR Dataset	Viral Pneumonia Lung Opacity COVID-19 Normal	X-Ray	1,345—Viral Pneumonia 6,012—Lung Opacity 3,616 – COVID-19 10,192 – Normal	[48]
CheXpert	Pneumonia and 13 others	X-Ray	Total – 2,24,316 4,576 – Positive	[49]
MIMIC-CXR	Pneumonia and 13 others	X-Ray	Total – 3,77,110 18,434 – Pneumonia	[50]
Covid19-Pneumonia-Normal Chest X-Ray Images	Pneumonia COVID-19 Normal	X-Ray	Total – 5,228 1,800—Pneumonia 1,626—COVID-19 1,802 – Normal	[51]
VinDr-CXR	Pneumonia and 27 others	X-Ray	Total—18,000 715—Pneumonia	[52]
COVID-QU-Ex	Viral/Bacterial Pneumonia COVID-19 Normal	X-Ray	Total—33,920 11,263—Viral or Bacterial Pneumonia 11,956—COVID-19 10,701—Normal	[53]
Covid19 Detection	Pneumonia COVID-19 Normal Fibrosis Tuberculosis	X-Ray	Total – 24,867 4,265—Pneumonia 3,616—COVID-19 11,800—Normal 1,686 – Fibrosis 3,500—Tuberculosis	[54]
Chest X-ray (Covid-19 & Pneumonia)	Pneumonia COVID-19 Normal	X-Ray	Total – 6,432 4,273—Pneumonia 576—COVID-19 1583—Normal	[55]

Access to private databases, which are often commercial and need authorization, is restricted. Publicly available datasets for prominent lung illnesses are presented [136]. Images of both pneumonia and healthy lungs can be found in the LDOCTCXR (http://data.mendeley.com/datasets/rscbjbr9sj/3) [42, 137] and RSNA pneumonia databases (https://www.kaggle.com/competitions/rsna-pneumonia-detection-challenge/data) [43].

The ChestX-ray8 dataset (https://www.kaggle.com/datasets/nih-chest-xrays/data) classifies eight lung diseases, such as pneumonia [44, 45], while the ChestX-ray14 dataset (https://www.v7labs.com/open-datasets/chestx-ray14) classifies 14 lung diseases using the same X-rays [46]. Researchers conducted a retrospective analysis on 155 patients with COVID-19 pneumonia treated with chest computed tomography in Rio de Janeiro, Brazil, between March and May 2020 (10.1590/0100-3984.2020.0133) [63].

According to the study, COVID-19 stimulates a distinct sort of pneumonia patients have discovered (10.17632/9xkhgts2s6.3) [47]. A specific kind of pneumonia known as viral pneumonia is discovered and recorded in this dataset (https://www.kaggle.com/datasets/tr1gg3rtrash/balanced-augmented-covid-cxr-dataset) [48]. According to the presented study, COVID-19 stimulates a distinct sort of pneumonia patients have found [47]. A specific kind of pneumonia known as viral pneumonia is discovered and recorded in this dataset [48].

Radiologist-labeled reference standard assessment sets and uncertainty labels are characteristics of CheXpert. The researchers evaluated various ways of addressing uncertainty and verified them on the assessment sets. The dataset includes 65,240 patients' chest radiographs, totaling around 2.5 million, that have been annotated for the presence of 14 chest radiographic findings. It has a labeler that can gather observations from free text radiological reports and use an uncertainty label to identify any uncertainties (10.48550/arXiv.1901.07031) [49]. 65,379 patients' X-ray scans are included in the 377,110 image MIMIC-CXR dataset. It comprises 253,714 frontal and 123,246 lateral view images (10.1038/s41597-019-0322-0) [50]. An open dataset of chest X-rays with radiologist annotations is called VinDr-CXR [52].

VinDr-CXR is a massive dataset with labels and more than 18,000 chest X-ray scan visuals made accessible to the public in DICOM format. All data, including images and findings, has been de-identified to safeguard patient privacy in the dataset. It comprises 715 pneumonia samples, accounting for 0.0397% of the dataset. Radiologists assigned labels to this dataset (10.1038/s41597-022-01498-w.) [138]. Ground-truth lung segmentation masks are included with the complete COVID-QU-Ex dataset (https://www.kaggle.com/datasets/anasmohammedtahir/covidqu) [53].

### Lung cancer

The reviewed study used databases for lung cancer that were open to the public to provide readers with pertinent information. The datasets for the diagnosis of lung cancer that are publicly available are listed in Table 7.

**Table 7:** Available lung cancerous datasets

Dataset Name	Lung Cancer Types	Modality	Number of Images	Reference
NIH Chest X-rays (ChestX-ray8)	Lung Nodule And 7 others	X-Ray	Total – 1,08,948 1,971—Lung Nodule 84,312 – No Findings	[44]
NIH Chest X-rays (ChectX-ray14)	Lung Nodule And 13 others	X-Ray	Total – 1,12,120 6,323—Lung Nodule 60,412 – No Findings	[46]
VinDr-CXR	Lung nodule and 27 others	X-Ray	Total – 18,000 586—Lung Nodule	[52]
SPIE-AAPM Lung CT Challenge	Benign and Malignant Lung Nodules	CT scan	Total – 22,489 37 – Benign Nodule 36—Malignant Nodule	[64]
Development of a Digital Image Database for Chest Radiographs with and Without a Lung Nodule (JSRT)	Lung Nodule Normal	X-Ray	Total—247 100—Malignant Nodules 54—Benign Nodules 93—Without Nodule	[56]
National Lung Screening Trial (NLST)	Lung Cancer	CT scan	Total—75,000 With and Without Nodule – 28,000	[65]
NSCLC-Radiomics	NSCLC	CT scan	Total—52,073	[66]
Cancer Moonshot Biobank—Lung Cancer Collection (CMB-LCA)	Lung Cancer	CT scan	Total—20,918	[67]
CT Ventilation as a functional imaging modality for lung cancer radiotherapy (CT-vs-PET-Ventilation-Imaging)	Lung Cancer	4D CT scan & PET	Total—29,491	[68]
Lung-PET-CT-Dx	Lung Cancer	CT scan & PET	Total—251,135	[69]
QIN LUNG CT	NSCLC	CT scan	Total—3,954	[70]
4D-Lung	NSCLC	CT scan – 4D fan beam 4D cone beam	Total – 3,47,330	[71]
RIDER Lung CT	NSCLC	CT scan	Total—15,419	[72]
RIDER Lung PET-CT	Lung Cancer	CT scan & PET	Total – 2,69,511	[73]

*NSCLC* Non-Small Cell Lung Cancer

While using the same X-ray instances, the ChestX-ray8 dataset classifies eight lung diseases, including the detection of lung nodules [44], while the ChectX-ray14 dataset classifies 14 lung disorders [46]. VinDr-CXR comprises 586 lung nodule samples, accounting for 0.0325% of the dataset [138]. The LUNGx Challenge will provide participants with a slight opportunity to contrast their diagnostic classification methods for 73 benign and malignant lung nodules (10.7937/K9/TCIA.2015.UZLSU3FL) [64]. The Japan Society of Radiological Technology has generated a dataset for lung nodule image classification (http://imgcom.jsrt.or.jp/minijsrtdb/) [56].

The NLST CT scan image collection, which comprises over 200,000 image series from 75,000 CT tests, was compiled by more than 25,000 individuals. The cancer data access system (CDAS) provided access to a subset of lung cancer images that contained around 28,000 images from approximately 3,700 individuals (https://cdas.cancer.gov/learn/nlst/images/) [65]. Four hundred twenty-two individuals with NSCLC are featured in the collection of images. A prognostic radiomic signature was created using the dataset (Lung1) that was reported (10.7937/K9/TCIA.2015.PF0M9REI10.7937/K9/TCIA.2015.PF0M9REI) [66]. Imaging data from the cancer moonshot biobank (CMB) is being made accessible in conjunction with the release of clinical and genetic data from the CMB initiative. CMB is a program of the National Cancer Institute that supports ongoing and upcoming studies into cancer research programs (10.7937/3CX3-S132) [67]. Lung cancer patients underwent a variety of diagnostic procedures, including an exhale or inhale breath-hold CT (BHCT), free-breathing four-dimensional CT (4DCT), and Galligas PET ventilation (10.7937/3ppx-7s22) [68]. The lung cancer patient's CT and PET-CT DICOM images are included in the database and XML annotation records (10.7937/TCIA.2020.NNC2-0461) [69]. Patients' CT scans were collected at the H. Lee Moffitt Cancer and Research Institute, which had NSCLC with a mix of stages and histology, and the QIN associates received the data for research objectives (10.7937/K9/TCIA.2015.NPGZYZBZ) [70]. Images collected during chemoradiotherapy for 20 patients with locally advanced NSCLC are included (10.7937/K9/TCIA.2016.ELN8YGLE) [71].

Assessing a Variety of Malignant, Unidimensional, Bidimensional, and Volumetric Parameters with CT Scans in Lung Cancer Patients, a collection of lung CT scans called the reference image database to evaluate therapy response (RIDER) was produced (10.7937/k9/tcia.2015.u1×8a5nr) [72]. To simplify the operations of the RIDER PET/CT subgroup, the RIDER lung PET/CT collection was shared (10.7937/K9/TCIA.2015.OFIP7TVM) [73].

### COVID-19

The datasets for the diagnosis of COVID-19 that are publicly available are listed in Table 8. The creators integrated 15 publicly available COVID-19 chest X-ray image datasets to build the curated COVID-19 posterior-anterior lung radiography imaging database [47]. Its four categories were the balanced augmented COVID CXR dataset, COVID-19, viral pneumonia, lung opacity, and normal.

**Table 8:** Available COVID-19 datasets

Dataset Name	COVID-19 Types	Modality	Number of Images	Reference
Curated Dataset for COVID-19 Posterior-Anterior Chest Radiography Images (X-Rays)	Bacterial Pneumonia Viral Pneumonia COVID-19 Normal	X-Ray	Total – 9,208 3,001—Bacterial Pneumonia 1,656—Viral Pneumonia 1,281—COVID-19 3,270—Normal	[47]
Balanced Augmented COVID CXR Dataset	Viral Pneumonia Lung Opacity COVID-19 Normal	X-Ray	Total – 21,165 1,345—Viral Pneumonia 6,012—Lung Opacity 3,616 – COVID-19 10,192—Normal	[48]
COVID-QU-Ex	COVID-19 Viral or Bacterial Pneumonia Normal	X-Ray	Total—33,920 11,956—COVID-19 11,263—Non-COVID infections 10,701—Normal	[53]
Covid19 Detection	COVID-19 Pneumonia Normal Fibrosis Tuberculosis	X-Ray	Total – 24,867 3,616—COVID-19 4,265—Pneumonia 11,800—Normal 1,686 – Fibrosis 3,500—Tuberculosis	[54]
Chest X-ray (Covid-19 & Pneumonia)	COVID-19 Pneumonia Normal	X-Ray	Total – 6,432 576—COVID-19 4,273—Pneumonia 1583—Normal	[55]
COVID-19-NY-SBU	COVID-19	CT & X-Ray	Total—5,62,376	[57]
CT Images in COVID-19	COVID-19	CT scan	Total—771	[74]
MIDRC-RICORD-1a	COVID-19	CT scan	Total—31,856	[75]
MIDRC-RICORD-1b	COVID-19	CT scan	Total—21,220	[76]
MIDRC-RICORD-1c	COVID-19	X-Ray	Total—1,257	[58]
COVID-19-AR	COVID-19	CT & X-Ray	Total—31,935	[59]
SARS-COV-2 Ct-Scan Dataset	COVID-19	CT scan	Total – 2,482 1,252 – Positive	[77]
COVID-XRay-5 K DATASET	COVID-19	X-Ray	Total—5,000	[60]
COVID-CT	COVID-19	CT scan	Total—349	[78]

It is a publicly available, significantly imbalanced chest X-ray dataset [48]. COVID-QU-Ex is the most comprehensive lung mask dataset ever created [53]. Combining multiple publicly accessible datasets created the COVID-19 detection dataset [54, 55]. Images from various organ locations and modalities are included in the collection (i.e., CXRs, CT scans, MRIs). For each patient, this collection comprises clinical information. Diagnoses, procedures, laboratory testing, and COVID-19-specific data values include clinical information [57]. A sample was taken within a day after the initial CT, resulting in a positive RT-PCR for SARS-CoV-2 in each subject. Conducted CT scans without contrast and converted DICOM images to NIfTI format (10.7937/TCIA.2020.GQRY-NC81) [74]. Across all COVID-positive thoracic CT imaging studies, pixel-level volumetric segmentation, including diagnostic captions by thoracic radiography general practitioners, was performed. This system of labels was put together with the help of other global consensus panels and COVID data annotation efforts (10.7937/VTW4-X588) [75]. 120 CT images of COVID-negative patients from four global sites make up the RSNA international COVID-19 open annotated radiology database (RICORD) version 1b. It gives access to a particular class of COVID-negative image collections (10.7937/31V8-4A40) [76]. Radiology subspecialists clinically annotated all COVID-positive X-ray studies using a labeling system based on COVID-19 reporting rules ( 10.7937/91ah-v663) [58]. The COVID-19-AR dataset has genome data and CT scans to understand better COVID-19 (10.7937/tcia.2020.py71-5978) [59, 139]. The COVID-XRay-5K dataset was produced using data gathered from two origins: The ChexPert Dataset is used for non-COVID or COVID-19 negative X-ray samples, whereas the Covid-Chestxray-Dataset is for COVID-19 positive X-ray samples (https://github.com/shervinmin/DeepCovid) [60]. In the COVID-CT collection, 4,63 patient CT scans are not included in the COVID-19 research. In addition, the COVID-19 collection contains 3,49 CT scans from participants who participated in the COVID-19 research (10.48550/arXiv.2003.13865) [78].

### Machine learning in pneumonia detection

An investigation of the several methodologies presently used for diagnosis and forecasting using a combination of ML and imaging methods is presented. Researchers from many areas, including ML and the medical sector, have looked at diagnosing and forecasting pneumonia.

The information was compiled from the final collection of articles describing the many sorts of ML techniques used and their findings, which are presented in Table 9.

**Table 9:** Machine learning and sub-fields in pneumonia diagnosis

Author/Ref.	Imaging Modality	Image Dataset Samples (with Classified Diseases)	ML Method	Performance Metrics(%)
Szepesi et al. [140]	X-Ray	4,273 – Pneumonia 1,583 – Normal 5,856 – Total Labeled Images	CNN + Modified Dropout	Accuracy—97.2 Recall – 97.30 Precision – 97.40 F1 Score – 97.40 AUC – 0.982
Avola et al. [141]	X-Ray	2,780 – Bacterial Pneumonia 1,493 – Viral Pneumonia 474 – COVID-19 1,583 – Normal 6,330 – Total	AlexNet, MnasNet, MobileNetv2, MobileNet v3, DenseNet, GoogleNet, ResNet50, ResNeXt, SqueezeNet, Wide ResNet50, VGG16, and ShuffleNet	Average F1 Score – 84.46
Liu et al. [142]	X-Ray	**Dataset 1:** 2,777 – Bacterial Pneumonia 2,838 – Viral Pneumonia 3,674 – COVID-19 11,768 – Normal 21,057 – Total **Dataset 2:** 2,777 – Bacterial Pneumonia 2,838 – Viral Pneumonia 3,665 – COVID-19 3,251 – Normal 12,531 – Total	**Multi-Branch Fusion Auxiliary Learning (MBFAL):** Auxiliary Learning method, and Prior-Attention Residual Learning (PARL) Architecture	**MBFAL Average:** Accuracy – 95.61
Srivastava et al. [143]	X-Ray	1,656—Viral Pneumonia 1,281—COVID-19 3,270—Normal 6,207 – Total	**Ensemble Model:** Ensemble DNN classifiers’ score based on Condorcet’s Jury Theorem **(CJT)** And Domain Extended Transfer Learning **(DETL)**	**CJT** - Accuracy – 98.22 Sensitivity – 98.37 Specificity – 99.79 **DETL** - Accuracy – 97.26 Sensitivity – 98.37 Specificity – 100
Qu et al. [144]	Infrared Thermal Images + RGB images	**Number of Subjects**: 30—Normal 28 – Pneumonia 58—Total	SVM KNN Decision Tree Gaussian Naïve Bayes classifier LDA, QDA	**Binary Classification:** Accuracy – 93.00
Singh et al. [145]	X-Ray	1,345—Viral Pneumonia 371—COVID-19 1,341—Normal 3,057—Total	Hybrid Social Group Optimization algorithm + Support Vector Classifier	Accuracy—99.65
Chowdhury et al. [146]	X-Ray	423—COVID-19 Pneumonia 1,485—Viral Pneumonia 1,579 – Normal 3,487—Total	**Three Shallow Networks:** MobileNetv2, SqueezeNet, and ResNet18 **Five Deep Networks:** Inceptionv3, ResNet101, CheXNet, VGG19, and **DenseNet201**	**Binary Classification** (Normal, Pneumonia) - Accuracy—99.70 Sensitivity – 99.70 Precision – 99.70 Specificity – 99.55 **Multi Classification** – Accuracy—97.90 Sensitivity – 97.95 Precision – 97.90 Specificity – 98.80
Wong et al. [147]	CT Scan (2D/3D)	4,017—Viral Pneumonia 7,766—Bacterial Pneumonia 3,443—Mycoplasma Pneumonia 10,687—COVID-19 11,666 – Normal 37,579—Total	CNN: Multi-Scale Attention Network (**MSANet**)	Accuracy—97.46 Recall – 96.18 Precision – 97.31 F1 Score – 96.71 Macro-Average AUC—0.9981
Ukwuoma et al. [148]	X-Ray	**Binary Classification** (Mendeley Dataset) – 4,290—Viral Pneumonia 3,834 – Normal 8,124 – Total **Multi Classification** (Chest X-ray Dataset) - 5,000—Viral Pneumonia 5,000—Bacterial Pneumonia 5,000 – Normal 15,000—Total	Ensembled CNN + Transformer Encoder Method **Ensemble A** (DenseNet201, VGG16, GoogleNet) **Ensemble B** (DenseNet201, InceptionResNetV2, Xception)	**Binary Classification** (Normal, Pneumonia) - Accuracy – 99.21 F1 Score – 99.21 **Multi Classification** Accuracy – 98.19 F1 Score – 97.29 **Ensemble Binary Class** **Ensemble A -** Accuracy – 97.22 F1 Score – 97.14 **Ensemble B -** Accuracy – 96.44 F1 Score – 96.44 **Ensemble Multi-Class** **Ensemble A -** Accuracy – 97.20 F1 Score – 95.80 **Ensemble B -** Accuracy – 96.40 F1 Score – 94.90
Kusk et al. [149]	X-Ray	4,273—Viral and Bacterial Pneumonia 1,583 – Normal 5,856 – Total	CNN + Gaussian noise (Five Gaussian Noise Levels)	Accuracy – (96.80—97.60) Sensitivity – (96.90—98.20) Specificity – (94.40—98.70)
Li & Li [150]	X-Ray	2,530 – Bacterial Pneumonia 1,345 – Viral Pneumonia 797 – COVID-19 5,510—Healthy 10,182 – Total	17 CNNs (AlexNet, GoogleNet, Vgg16, ResNet18, SqueezeNet, MobileNetv2, Inceptionv3, DenseNet201, Xception, Vgg19, Places365GoogleNet, InceptionResNetv2, ResNet50, ResNet101, NASNetMobile, NASNetLarge, ShuffleNet)	Distinguishing Covid-19 Pneumonia from Bacterial Pneumonia - (Accuracy – 99.85) Normal Lung Images (Accuracy – 100) Viral Covid-19 Pneumonia (Accuracy – 99.95)
Bhandari et al. [151]	X-Ray	4, 273 – Pneumonia 576—COVID-19 700 – TB 1583 – Normal 7,132 – Total	CNN + XAI + Grad-CAM, Local Interpretable Modelagnostic Explanation (LIME), and SHapley Additive exPlanation (SHAP)	Overall Accuracy – 95.94 **Average -** Specificity – 95.71 ± 1.55 Sensitivity – 95.50 ± 1.72 F1 Score – 96.53 ± 0.95
Khaniabadi et al. [152]	CT Scan	100 – Pneumonia 100 – COVID-19 100—Healthy 300 – Total	ML Algorithms: SVM, KNN, Decision Tree, Naïve Bays, Bagging, **Random Forest, and Ensemble Meta voting**	**Random Forest, and Ensemble Meta voting -** Accuracy(RF) – 0.94 ± 0.031 Accuracy(EM) – 0.92 ± 0.034 Sensitivity(RF) – 0.90 ± 0.056 Sensitivity(EM)—0.90 ± 0.078 Specificity(RF) – 0.95 ± 0.020 Specificity(RF) – 0.95 ± 0.010 AUC—0.98 ± 0.010 AUC—0.92 ± 0.043
Ascencio-Cabral et al. [153]	CT Scan	2,946—Community Acquired Pneumonia 7,593 – COVID-19 6,893 – Non-COVID-19 17,432 – Total	Transfer Learning: ResNet-50, ResNet-50r, DenseNet-121, MobileNet-v3, and CaiT-24-XXS-224 (CaiT) Transformer	**ResNet-50’s –** Accuracy – 98.00 Balanced Accuracy – 98.00 F1 Score – 98.00 F2 Score – 98.00 MCC – 98.00 Sensitivity – 98.00 Specificity – 98.00

*LDA* Linear Discriminant Analysis, *QDA* Quadratic Discriminant Analysis, *XAI* Explainable Artificial Intelligence, *Grad-CAM* Gradient-weighted Class Activation Mapping, *CJT* Condorcet's Jury Theorem, *DETL* Domain Extended Transfer Learning

The dropout convolutional network proposed by Szepesi et al. was trained and evaluated on 5856 tagged images. A convolutional layer with a unique dropout was part of the proposed architecture, along with a batch normalization layer, an activation layer, and a pooling layer. The researchers evaluated the test performance of the proposed model at several different dropout rates, including 10%, 20%, 30%, 40%, and 50%, and the results showed that the 40% dropout rate was the most successful. Their retrospective analysis included one-to-five-year-old children with anterior–posterior (AP) X-rays [140].

Twelve ML models had already undergone training —AlexNet, DenseNet, GoogleNet, MnasNet, MobileNetv2, MobileNetv3, ResNet50, ResNeXt, ShuffleNet, SqueezeNet, VGG16, and Wide ResNet50—were modified and used to predict X-rays of healthy people and those with pneumonia symptoms that could be caused by either a virus or bacteria. It was done to distinguish between healthy people and those who could have pneumonia symptoms caused by viral or bacterial agents. To provide an informative analysis of model classification, we presented additional experiments to evaluate the resilience of each model. These experiments utilized 50%, 20%, and 10% of the training data. It gave an average f1-score of 84.46% when trying to tell the difference between the four classes [141].

Multi-branch fusion auxiliary learning (MBFAL) is a suggested approach for analyzing CXR images to diagnose pneumonia. The proposed MBFAL approach is comprised of ResNet34 and ResNet18, which were previously trained on the ImageNet dataset. The training was conducted using the ResNet18 and ResNet34 networks, the auxiliary learning method, the prior-attention residual learning (PARL) network, and the MBFAL technique. This technique is based on supplementary learning and verifying fit sets using an auxiliary database. This is performed in combination with the PARL structure and feature fusion approach. A multi-branch CNN achieved classification, and the fusion of losses during network training involved using an MLP [142].

Based on Condorcet’s Jury Theorem (CJT), the unique method calculated classifier voting ensemble scores. The studies showed, with the assistance of CJT, that including a model in the pool of voters would increase the chance that the majority vote would be correct if the model in question were more accurate than the other models in the pool. In addition to this, a different unique domain extended transfer learning (DETL) ensemble classifier was constructed as a soft voting ensemble technique. This model has been compared against a CJT-based ensemble classifier to determine which is superior. Because of the large number of classifier votes in ensemble learning, it is necessary to consider each vote and significant voting. The winning class in majority voting is the one with the most votes. However, a higher number of votes does not necessarily increase the chances that the final verdict will be correct [143].

A portable, quick thermal imaging system proposed with image-processing algorithms and ML analysis for pneumonia diagnosis. A smartphone-attached portable thermal imager recorded RGB and infrared images from the back of each issue. Pneumonia patients' back lung mapping skin temperature increased substantially, which may help diagnose them. The obtained images were then automatically processed to extract several spatial and structural attributes that can accurately differentiate between normal individuals and patients suffering from pneumonia. The procedure for detection is as follows: determining the highest temperature in each thermal image indicating the pulmonary area on the accompanying RGB image, Identifying the spot on the thermal image after obtaining the temperature in the area of overlap, Calculating the high-temperature indices Utilizing principal component analysis (PCA) to analyze the indices. In addition, thermal imaging was used for the diagnosis and treatment evaluation of pneumonia in this investigation [144].

The Hybrid Social Group Optimization (HSGO) method extracted relevant and critical features from CXR images. Several classifiers categorized CXR images. The social group optimization (SGO) approach with enhancements, HSGO, chooses the optimal features from a feature collection. A wrapper-based method enables HSGO to locate the optimal feature set more efficiently [145].

In conjunction with image augmentation, transfer learning is employed in training and validating multiple pre-trained deep CNNs. The neural networks were learned to categorize using two distinct methods: first, binary classification, and second, multi-classification with and without image augmentation. The performance of deep networks was demonstrated to be superior to that of shallow networks when both types of networks were trained using image augmentation. Image augmentation training showed that DenseNet201 outperformed other CNN networks. DenseNet-based CheXNet outperformed other networks without image augmentation. Deeper DenseNet supersedes CheXNet on a huge augmented dataset [146].

The multi-scale attention network (MSANet) approach may automatically prioritize unique statistical features and multi-scale characteristics of pneumonia detection to enhance classification. Four modules—lung segmentation, spatial pyramid decomposition, multi-scale feature extraction, and classification—make up this approach. Community-acquired pneumonia (CCAP) dataset is a public, multiclass CT scan dataset that includes four different types of pneumonia [147].

Combining the capabilities of Ensemble CNN with the Transformer Encoder method produces the proposed fusion methodology. Ensemble A hybridizes DenseNet201, VGG16, and GoogleNet, whereas Ensemble B is a hybridization of DenseNet201, InceptionResNetV2, and Xception. The ensemble backbone retrieves significant features from the input X-ray images using two independent ensemble methods. On the other hand, the MLP self-attention mechanism is used to make the Transformer Encoder for accurate diagnosis [148].

The specified research aimed to develop and assess CNNs for identifying pneumonia based on CXR images with varying image noise levels. Six classification tasks were designed for five levels of Gaussian noise. The images had Gaussian noise added to them with a zero mean, and there were five different levels of image noise variance, which corresponded to reducing exposure levels. CNN's analysis of the various datasets found no significant loss in performance when comparing the original input dataset to the five datasets with varying noise levels [149].

Li and Li created a new voting technique to combine 17 CNNs and use them to construct our AI models for data fitness optimization to prove that the 17-CNN approach is better than any individual CNN approach. Classifier A compares patients with pneumonia to those without; classifier B contrasts viruses and bacteria; classifier C differentiates between COVID-19 and other viruses; classifier D does the same for COVID-19 and bacteria; and classifier E compares COVID-19 and healthy individuals. To use transfer learning, CNNs are kept the same during the first training on the secondary domain. Only the layers that come after that are changed [150].

The model that is being proposed is a combination of a CNN and explainable AI. Grad-CAM, LIME, and SHAP are used to analyze and describe the information for more understanding. The extraction of convolutional features is used to gather high-level, object-based data. Next, the CNN model's black-box technique is assessed utilizing shapely information from SHAP, predictive results from LIME, and a heat map from Grad-CAM [151].

A two-step ML-based diagnostic and predictive model was designed. Lungs were segmented using DL-based segmentation. One hundred seven features were retrieved, including contour, histograms, and high-order texture features, and accompanied by various methods for selecting features, which were also utilized. GLCM, GLRLM, GLDM, GLSZM, and NGTDM were used to compute the features. The classifications of pneumonia, COVID-19, and healthy and severe, moderate, and mild score indices were calculated using random forest and meta-voting [152].

Five architectures for deep learning ResNet-50, ResNet-50r, DenseNet-121, MobileNet-v3, and CaiT-24-XXS-224 (CaiT) transformers are used for transfer learning. Researchers conducted twenty examinations with ten repeats, evaluating the classifiers' efficiency by applying the Friedman-Nemenyi test. The boot-strapping method was used to make confidence intervals, and then the Friedman–Nemenyi paired post hoc test was used to compare models. ResNet-50 architectures are statistically guaranteed to be robust enough to diagnose pneumonia in a multiclass environment [153].

### Machine learning in lung cancer detection 

Throughout this part, researchers have investigated the various techniques or procedures currently employed for identifying lung cancer, and these approaches are addressed. The findings of research studies examining the identification and prediction of lung cancer are summarized in Table 10.

**Table 10:** Machine learning and sub-fields in lung cancer

Author/Ref.	Imaging Modality	Image Dataset Samples (with Classified Diseases)	ML Method	Performance Metrics(%)
Sekeroglu et al. [154]	CT Scan	**LIDC/IDRI** – 100—Annotated Nodules 604 – Total Nodules & non-nodules (diameter ≥ 3 mm)	Multi-Perspective Hierarchical Deep Fusion Learning Approach	Accuracy – 91.20 Specificity – 87.00 Sensitivity – 95.00 False Positive/scan—0.4
Donga et al. [155]	CT Scan	**LIDC/IDRI** – 1018—Total	Modified Gradient Boosting Algorithm	Accuracy – 95.67 Precision – 95.70 Recall – 91.00 F1 Score – 94.10
Khehrah et al. [156]	CT Scan	**LIDC** – (~ 250–350)—Nodule’s Images of 70 lung Scans	Otsu method + SVM	Accuracy—92.00 Sensitivity – 93.75 Specificity – 91.18 Precision – 85.19 FPI – 0.13 FPE – 0.22 MCC – 0.8385
Ausawalaithong et al. [157]	X-Ray	**JSRT –** 100 – Malignant ( +) 147 – Benign and Normal (-) 247—Total **ChestX-ray14 -** 6,282 – Positive ( +) 1,05,197 – Negative (-) 1,11,479—Total	Transfer Learning - Base Model – Densenet-121 Retrained Model – A (On ChestX-ray14) Retrained Model – B (On JSRT) Retrained Model – C (On ChestX-ray14 + JSRT)	**Retrained Model—C** **Mean** - Accuracy—74.43 + 6.01 Specificity—74.96 + 9.85 Sensitivity – 74.68 + 15.33
Chen et al. [158]	CT Scan	10,000—Total	Manual SegNet Deeplab v3 VGG 19	Accuracy – 92.50 Sensitivity—98.33 Specificity – 86.67 Overlap Rate-95.11
Nanglia et al. [159]	Low-Dose CT Scan (LDCT)	500—Total	**Feature Extraction** – SURF + Genetic Algorithm **Classification** -SVM + Feed Forward Back Propagation Neural Network	Overall Accuracy – 98.08 Precision—98.17 Recall—96.50 F-measure – 97.00
Alshmrani et al. [160]	X-ray	20,000 – Lung Cancer 3,615 – COVID-19 5,856 – Pneumonia 6,012—Lung opacity 1,400 – Tuberculosis 10,192—Normal 80,000—Total	VGG19 + 3 Blocks of CNN	Accuracy – 96.48 Precision – 97.56 Recall – 93.75 F1 Score – 95.62 AUC – 99.82
Heuvelmans et al. [161]	CT Scan	**NLST** - 205—Malignant 2,106 – Total Lung Nodules	Lung Cancer Prediction CNN (LCP-CNN)	Sensitivity – 99.00 AUC—94.50
Rahouma et al. [162]	CT Scan	30 – NSCLC 20 – Benign 50 – Total Lung Nodules	Polynomial Neural Network (PNN)	Accuracy—96.66 Sensitivity – 95.00
Bilal et al. [163]	X-ray	250 – Normal 320 – Benign 320 – Malignant 910 – Total	VGGNet, ResNet, GoogLeNet AlexNet, InceptionNet-V3 + Improved Gray Wolf Optimization and InceptionNet-V3	Accuracy – 98.96 Sensitivity—100.00 Specificity – 94.74
Torres et al. [164]	CT Scan	09—Benign 51—Malignant 60 – Total Lung Nodules	**Nodule Extraction** – Otsu thresholding and morphological operations + GLCM + t-test **Classification**—Feed-Forward Neural Network	Accuracy – 96.30 Sensitivity—100.00 Specificity – 83.00 F1 Score – 97.67 AUC – 94.00
Hussain et al. [165]	MRI	377 – NSCLC 568 – SCLC 945 – Total Lung Nodules	**(I)** Texture features using SVM polynomial **(II)** Image Adjustment using SVM RBF and Polynomial **(III)** Contrast stretching at threshold of (0.02, 0.98) using SVM RBF and Polynomial **(IV)** Gamma Correction at gamma value 0.9	**(I)** Sensitivity = 100 Specificity = 99.72 Accuracy = 99.89 **(II), (III), and (IV) -** Sensitivity = 100 Specificity = 100 Accuracy = 100
Kuo et al. [166]	CT Scan	273 – GGO 120 – Part Solid 274 – Solid 667 – Total Lung Nodules	**Preprocessing** – Adaptive Wiener filter **Lung Segmentation**—Fast Otsu & Edge Search Method **Nodule Enhancement**—Gray Level Adjustment **Candidate Detection**- Fast Otsu Method **Classification**—SVM	**Total** Sensitivity—92.05 **Small Nodules** (5 mm–9 mm) - Sensitivity—93.73 **GGO** – Sensitivity—93.02
Singh et al. [167]	CT Scan	6,910 – Benign 8,840 – Malignant 15,750—Total Lung Nodules	**Feature Extraction** – GLCM + Statistical Method **Classification** -KNN, SVM, DT, RF, MLP, Naïve Bayes, Gradient Descent	Accuracy—88.55 Sensitivity – 89.84 Precision – 86.59 F1 Score – 87.35

*LIDC* Lung Image Database Consortium, *IDRI* Image Database Resource Initiative, *FPI* False Positive per Image, *FPE* False Positive per Exam, *JSRT* Japanese Society of Radiological Technology, *MCC* Matthews correlation coefficient, *NLST* National Lung Screening Trial, *NSCLC* Non-Small-Cell Lung Carcinoma, *SCLC* Small Cell Lung Carcinoma, *RBF* Radial Basis Function, *GGO* Ground Glass Opacity

Researchers constructed three distinct hierarchical deep-fusion learning models to identify lung nodules from CT scans. The completed model includes MPF, SFMPF, and MFMPF, which stand for multi-perspective, single-feature, and multi-feature, respectively. The MPF model has three hierarchical classification levels based on multi-perspective deep fusion. SFMPF is a model for image-feature-based hierarchical deep fusion learning. Using bilateral, trilateral, Gabor, and LOG-filtered images, four distinct feature-image-based model architectures are investigated. Combining the outputs of the four SFMPF models yields the MFMPF [154].

Images from CT scans are preprocessed to improve quality. Next, the lung nodule regions are segmented using a random walker algorithm based on user-provided seeds. Then, the LBP and the Riesz wavelet transform are used to collect the intensities and texture features. The improved gradient boost classification model was developed and evaluated to identify nodules as malignant or benign using the managed features [155].

The identification of lung nodules in CT images has been reported using statistical and shape-based parameters. Lung segmentation was achieved using a histogram-based threshold approximation approach. Extraction of nodule features utilizing statistical and shape-based techniques and an algorithm for detecting round or almost round shapes to identify circular ones. For processing purposes, DICOM images are converted to PNG format. DICOM is a storage and transmission standard for medical images. Digital images that may result in image quality deterioration The testing phase of the SVM classifier produced superior results [156].

The 121-layer CNN, DenseNet-121, and the transfer learning scheme are potential classification methods. Transfer learning was used and considered due to the issue of a minimal dataset in the JSRT dataset. The first way to classify transfer learning is based on whether or not it involves nodule formation. The next thing that needs to be done is to ascertain whether or not the nodule in concern is malignant [157].

The CT scan was manually segmented and then analyzed using a convolutional neural network. Even though the segmentation results based on DeepLab v3 and VGG-19 are better than those of the artificial segmentation, the testing revealed that both SegNet and the artificial segmentation findings are the nearest to the benchmark and almost overlap. Pathological evaluation revealed that 120 patients had benign lung nodules, whereas the same number of patients had benign lung nodules discovered by SegNet within the same period [158].

The suggested Block-PP employed morphological processes in conjunction with fuzzy logic to complete the lung segmentation. The SURF approach and the genetic algorithm are used in conjunction with the suggested Block FE–O to carry out the processes of feature extraction and optimization, respectively. The optimized or chosen feature set was then transmitted to the proposed Block-HB using the SVM and the feed-forward-back-propagation-neural-network (FFBPNN) [159].

Using the DL architecture for multiclass classification that was created, several illnesses, including pneumonia, were categorized. For classification, a VGG19 model that had already been trained was used. After that, three blocks of CNN were used to pull out features, and a fully connected layer was used for classification [160].

CT scan images were employed in the training process of a lung cancer prediction CNN (LCP-CNN) that had been developed to assign a malignancy score to each pulmonary nodule. Training for the LCP-CNN was carried out with the assistance of the NLST dataset. The LCP-CNN rule-out test was developed to determine benign nodules while keeping a high degree of sensitivity intact. This was accomplished by using malignancy score thresholds. During the procedure of defining the rule-out criteria, an eight-fold cross-validation method was employed [161].

The presented method consists of four stages: first, image preprocessing using the Gabor and Kuwahara filters.

Secondly, image segmentation was accomplished using Chan-Vese active contour modeling to exclude minor perturbations to previously discovered nodules, like small fragments wrongly identified as nodules. In this instance, little nodules were found by segmenting the lung region using a region-growing algorithm. The third step was feature extraction, which generated features using the DWT at one, two, and three decomposition levels. Finally, following a comparison of the output features, the polynomial neural network (PNN) categorization algorithm is trained to differentiate benign from malignant nodules based on the output feature that was determined to be the most accurate [162]. A hybrid method was proposed that used CNN models, the transfer learning approach, gray wolf optimization (GWO), and genetic algorithms (GA). A weighted filter was used to minimize the image noise, and an enhanced version of the Gray Wolf Optimization approach was carried out before the segmentation process, along with watershed modification and dilation procedures. The combination of improved Gray Wolf optimization and Inception-V3 (IGWO-IV3) increased overall performance. The IGWO uses GA to locate the most advantageous starting sites for the GWO [163].

A hybrid strategy for characterizing nodules in CT images by combining the features used to identify them with the extension of feed-forward networks. Researchers developed an embedding of nodules that are based on the statistical relevance of features for malignancy identification to reduce the amount of training data that was also required. Leveraging self-defined diagnostic performance measurements, a feed-forward network also optimizes its structure and hyper-parameters [164].

The research endeavored to enhance the quality of images of lung cancer by using and applying various imaging techniques, like image correction, gamma correction, contrast stretching, thresholding, and histogram equalization techniques. Features obtained by the GLCM to improve images and use and refine several robust machine learning classification approaches, like SVM with Gaussian, RBF, and polynomial kernels, decision trees, and naive Bayes [165].

An automated approach to identifying lung nodules using CT image processing methods is presented. The oval or circular form of the lung nodules' two-dimensional shape is used as the basis for the detection approach for the lung nodules. It is feasible to identify a lung nodule using four 2-dimensional features and then classify it using eleven 3-dimensional features. Nodule enhancement is the process of increasing the gray level of nodules. The method was applied to an image, which resulted in the lower brightness level of the image being amplified while the upper brightness level of the image remained unaltered [166].

Effective presentation of image preprocessing techniques such as denoising, thresholding, and morphology. Denoising and thresholding are done using Gaussian blur and Gaussian thresholding, respectively. The provided image is converted to grayscale and de-noised using Gaussian blur for image processing. After that, Otsu's technique and adaptive Gaussian thresholding altered the grayscale image. Form-based morphological procedures were then performed on the image. They also proposed a novel algorithm and image-processing approach. Texture features are retrieved utilizing statistical parameters and GLCM, which are applied to extract features from the segmented images with enhanced quality. A performance evaluation of seven ML-based classifiers for detection and classification is presented [167].

### Machine learning in COVID-19 detection

This section examines various COVID-19 diagnostic techniques and approaches presently in use. The information shown in Table 11 was derived from a compilation of publications describing the different ML approaches and their results.

**Table 11:** Machine learning and sub-fields in COVID-19

Author/Ref.	Imaging Modality	Image Dataset Samples (with Classified Diseases)	ML Method	Performance Metrics(%)
Wang et al. [168]	X-Ray	**COVIDx:** 13,975 – COVID-19 +	**COVIDNet:** Machine Driven Design Exploration: Projection-Expansion-Projection-Extension (PEPX) Architecture	Accuracy—93.30 Sensitivity – 91.00 Positive Predictive Value – 98.90
Keles et al. [169]	X-Ray	210—COVID-19 + 350—Viral Pneumonia 350—Normal 910—Total	**COV19-CNNet:** Feature Engineering—7 convolutional layers Classification—4 Dense Layer	Accuracy—94.28 Specificity—96.94 Sensitivity—94.33 F1-score—94.20
			**COV19-ResNet:** (Based on ResNet)	Accuracy—97.61 Specificity – 98.72 Sensitivity – 97.61 F1-score – 97.62
Ohata et al. [170]	X-Ray	**Dataset-A**: 194—COVID-19 + 194 – Healthy 388—Total	Transfer Learning with MobileNet + Linear SVM	Accuracy—98.46 F1-score—98.46 FPR – 1.026
		**Dataset-B**: 194—COVID-19 + 194—Healthy 388—Total	Densenet201 + MLP	Accuracy—95.64 F1-score—95.63 FPR – 4.103
Singh et al. [171]	X-Ray	**Dataset-A**: 573—COVID-19 + 573—Normal 573 – Pneumonia 1,719—Total **Dataset-B**: 1,519—COVID-19 + 1,519—Normal 1,519—Pneumonia 4,557—Total **Dataset-C**: 573—COVID-19 + 1,600—Normal 1,600 – Pneumonia 3,773—Total	**COVIDScreen (Pruned Ensemble Learning framework)**: **Base Learners** – VGG-19, VGG-16, DenseNet-121, DenseNet-169, ResNet-50 **Meta learner** – Naïve Bayes + GAN	Accuracy—98.67 Precision – 100.00 Recall – 100.00 F1-score – 100.00 Kappa score—0.98
Iqbal et al. [172]	X-Ray	**Dataset-1:** 284—COVID-19 + 310—Normal 330—Pneumonia Bacterial 327—Pneumonia Viral 1,251—Total **Dataset-2:** 157—COVID-19 + 500—Normal, 500—Pneumonia, 1,157—Total	**CoroNet:** Xception (An Extreme Version of Inception Model – 71 Layer), Flatten, Dropout, Dense	**CoroNet on Dataset-1:** Average - Precision- 93.17 Recall—98.25 Specificity – 97.90 F1-Score—95.61 Accuracy 4 class—89.60 Accuracy 3 class – 95.00 Accuracy 2 class—99.00 **CoroNet on Dataset-2:** Overall Accuracy- 90.21 Precision – 97.00 Recall – 89.00 Specificity—99.6 F-measure – 93.00 **Overall 3 and 4 Class CoroNet:** Accuracy-89.60
Madaan et al. [173]	X-Ray (Frontal Postero- anterior)	**Dataset-1:** 196—COVID-19 + **Dataset-2:** 1,583—COVID-19-	**XCOVNet:** Convolution (First – 32, Second – 64, Third—128) + ReLu + Adam Optimizer	Accuracy—98.44
Das et al. [174]	X-Ray (Frontal)	**Generated:** 538—Class 0 (COVID-19 +) 468—Class 1 (COVID-19-) 1,006—Total	**Ensemble method:** Combination of InceptionV3, Resnet50V2 and Densenet201	Accuracy- 91.62 Sensitivity– 95.09 Specificity—88.33 F1-score—91.71 AUC—91.71
Hussain et al. [175]	X-Ray	**COVID-R:** 2,843—COVID19 + 3,108—Normal 1,439 – Pneumonia (Viral + Bacteria) 7,390—Total	**CoroDet model(22-layer):** 9 Conv2d layers, 9 maxpool2d layers, 1 Flatten, 2 dense, 1 LeakyReLu	**2 class classification:** Accuracy—99.12 **3 class classifications:** Accuracy—94.20 **4 class classification:** Accuracy—91.20
Rahman et al. [176]	X-Ray	**COVQU:** 3,616—COVID19 + 8,851—Normal 6,012 – Non-COVID Total – 18,479 CXR	**Lung segmentation**: Modified U-net **Classification:** 7 Deep CNN model (ResNet18, ResNet50, ResNet101, InceptionV3, DenseNet201, and ChexNet and a shallow CNN model)	**Lung segmentation:** Accuracy—98.63 Dice Score – 96.94 **Classification:** Accuracy—96.29 Sensitivity- 97.28 F1-score—96.28
Narin et al. [177]	X-Ray	**Dataset-1:** 341—COVID-19 + 2,800—Normal 3,141—Total **Dataset-2:** 341—COVID-19 + 1,493—Viral pneumonia 1,834- Total **Dataset-3:** 341- COVID-19 + 2,772 – Bacterial pneumonia 3,113—Total	InceptionV3, ResNet50, ResNet101, ResNet152, Inception-ResNetV2	**Binary Classification:** Accuracy: **Dataset-1:** COVID-19—96.10 **Dataset-2:** COVID-19—99.50 **Dataset-3:** COVID-19—99.70
Gaffari Celik [178]	CT scan & X-Ray	**CT scan images:** 1,601– COVID-19 + 1,693 – Normal 3,294 – Total **X-Ray images**: 3,616 – COVID-19 + 10,192 – Normal 6,012—Lung Opacity 1,345—Viral pneumonia 21,165 – Total	**CovidDWNet:** Feature Reuse Residual Block and Depth-wise Dilated Convolutions + Gradient Boosting Architecture	**Binary Class:** (CT Images) Accuracy – 100.00 (Application 1) Accuracy – 99.84 (Application 2) **Multi-Class: (**X-Rays) Accuracy – 96.81 (Application 3) Multi-Class (CT and X-Rays) Accuracy – 96.32 (Application 4)
Gozes et al. [179]	CT scan	829—COVID-19 + 1,036—COVID-19- 1,865—Total	**Lung Segmentation**: Proposed U-net with VGG-16 base encoder **Classifier**: ResNet-50	AUC – 94.80 (95% CI: 0.912–0.985)
Ahuja et al. [180]	CT scan	349—COVID19 + 397 – NonCOVID19 746—Total	**Augmentation:** Rotation + Translation + Shearing + SWT **Transfer Learning:** SqueezeNet, ResNet18, ResNet50, ResNet101	**Binary Class: ResNet18** Accuracy—99.40 Sensitivity- 100.00 Specificity – 98.60 F1-score – 99.50 NPV – 100.00
Silva et al. [181]	CT scan	**SARS-CoV-2 CT scan:** 1,252—COVID19 + 1,230 – NonCOVID19 2,482—Total **COVID-CT:** 349—COVID19 + 463 – NonCOVID19 812—Total	**EffiecintCovidNet:** Transfer Learning - Base Learner—EfficientNet B0 Architecture	Accuracy—98.99 Sensitivity – 98.80 Positive Prediction – 99.20

*MLP* Multi-Layer Perceptron, *FPR* False Positive Rate, *GAN* Generative Adversial Network, *NPV* Negative Predictive Value

COVIDNet is a deep CNN designed to detect COVID-19 in lung X-rays. They created the COVIDx dataset, which consists of five datasets that are accessible online. The projected COVID-Net had already been trained on the ImageNet and then trained on the COVIDx dataset. Training settings included a learning rate 2e4, 22 epochs, 64 batches, a factor of 0.7, and a patience setting of 5. The neural network architecture provided by the COVID-Net framework is the only one of its type to provide a compact projection-expansion-projection-extension (PEPX) architecture.

This architecture improves representational capacity while significantly reducing computational complexity [168].

Two diagnostic inference engines, COV19-CNNet and COV19-ResNet, are employed for COVID-19 diagnosis. Both architectures were developed from scratch without the use of a pre-trained DL model. AI-based inference engines can transform X-ray equipment into valuable testing equipment for diagnosing COVID-19 using specified DL methods. In contrast to earlier research in the area, inference engines were constructed from the ground up, utilizing novel deep neural networks and no preexisting systems. COV19-CNNet and COV19-ResNet are the two engine designations. The COV19-CNNet employs a CNN architecture, whereas the COV19-ResNet employs a ResNet structure. They focused their study on the complexity of classifying COVID-19 into multiple groups [169].

Transfer learning and classification utilizing a linear SVM classifier and MobileNet architecture accomplish automatic X-ray image detection. Images of healthy individuals were used for datasets A and B, but COVID-19 images remained unchanged. Multiple CNN architectures can extract features from X-ray images due to subsequent training on ImageNet. CNNs are combined with MLP, KNN, and Naïve Bayes [170].

Image enhancement, segmentation, a customized stacking ensemble model with four CNN base-learners (DenseNet-121, DenseNet-169, VGG-16, VGG-19, and ResNet-50), and Naive Bayes as a meta-learner are all part of the "COVIDScreen" developed model for classifying lung X-rays. After the preprocessing stage, which included histogram equalization with CLAHE and image segmentation with U-Net techniques [171], the dataset was 6% more accurate.

The researchers conducted four class classifications (Normal, COVID-19, Pneumonia Bacterial, and Pneumonia Viral) on various prepared datasets by using the suggested CoroNet model. Additionally, they did three class classifications of "normal," "COVID-19," and "pneumonia" on these datasets. The "CoroNet" suggested model was built on top of the Xception CNN architecture as its primary building block. The Inception design was extended to 71 layers to create the Xception architecture [172].

CNN was used to perform a two-phase X-ray image analysis process known as "XCOVNet" for COVID-19 detection. During the first step, the collection of X-ray pictures, of which fifty percent are positive for COVID-19 and the other fifty percent are normal, was preprocessed. In the second step, the neural network model was trained and fine-tuned to attain a classification accuracy of 98.44 percent. In this investigation, researchers used two chest X-ray imaging collections: Dataset-1 consists of 950 CXR images annotated with more than fifteen various types of illness discoveries with 196 COVID-19 CXRs. In contrast, Dataset-2 consists of 5856 CXR images with 1,583 COVID-19 CXRs classified as bacterial, viral, and normal pneumonia [173].

The researchers classified COVID-19 using a graphical user interface (GUI) tool they designed. They used many CNN models, including DenseNet 201, Resnet 50 V2, and Inception V3. Each model underwent meticulous instruction so that it would be able to provide accurate forecasts. After that, the technique for assembly is employed to attach the models [174].

The authors' proposed method, known by its acronym CoroDet, is comprised of an original 22-layer (9 Conv2d layers, 9 Maxpool2d layers, one flattened layer, two dense layers, and one leaky ReLu layer) CNN model. Multiple classifications were performed, including two, three, and four classes. During their study, they did 7390 scans in the COVID-R dataset they built [175].

The COVQU dataset consisted of 18479 CXRs of patients with normal lungs, lung capacity abnormalities associated with COVID-19, and lung capacity disorders unrelated to COVID-19. They introduced a modified version of the U-Net network for lung segmentation and classification that uses seven different CNN models: six deep CNN models (ChexNet, DenseNet201, InceptionV3, ResNet101, ResNet50, and ResNet18) and one shallow CNN model [176].

Five distinct CNN models were employed for three binary classifications as part of a deep transfer learning-based strategy. According to the research, the primary advantage of using transfer learning for data training is that it requires fewer data points. ResNet had the most remarkable accuracy of all the trained models in the research. For their investigation, they built multiple datasets using CXR images from several publicly available datasets [177].

The CovidDWNet approach uses a structure built on feature reuse residual blocks and depth-wise dilated convolutional component elements. Both of these components are convolutional in nature. Using the gradient boosting method, we obtained an estimate for the feature maps produced with the assistance of the COVIDWNet architecture. An efficiency increase of almost 7% was realized with the aid of the CovidDWNet + GB architecture in CT scans, while an efficiency improvement of approximately 4% was reached in X-ray imaging [178].

For patient-specific per-slice CT scan analysis, researchers recommended 2D processing. The processing is as follows: Step 1 helped them; 2D ROI segmentation acquired the lungs. Step two evaluates segment conditions using a 2D ROI classifier. Step three uses Grad-Cam, a multi-scale model, to create a localization map. The fourth step integrated all segment localization maps to create a 3D concatenated volume. Step five introduces the Corona-score biomarker and 3D volumetric scoring. Step six determines the severity of the illness. When a case is positive, the system provides a Corona score, used in research to assess severity [179].

Using transfer learning, developers developed a detection system. To achieve a higher level of accuracy, they suggested a stage-based detection strategy that included the following procedures: The first step required the augmentation of data; the second phase made use of a CNN model that had been pre-trained; and the third phase focused on the localization of anomalies in CT scan images [180].

Voting was the basis for a system that research suggested. Images are divided up into their respective categories with the use of a voting process in this approach. One can perform a cross-dataset evaluation to evaluate the robustness of the models by utilizing data from several different distributions [181].

## Methodical exploration

The significant concerns still in consideration:

- *Image Dataset Availability*: Since there is a need for imaging samples and datasets available, it might be challenging to acquire all of the information necessary to diagnose lung illness accurately.
- *Imbalanced Datasets*: Imbalance in the dataset can lead to inaccurate diagnosis, as DL solutions may overfit the majority or minority classes and fail to classify accurately.
- *Quality of Images*: Low-resolution or poor-quality images can yield inaccurate results when using ML solutions for lung disease diagnosis.
- *Unreliable data*: ML models rely highly on high-quality, consistent data, which can be hard to come by. Poor quality, incomplete, or inconsistent data can lead to an incorrect diagnosis.
- *Bias in data*: Healthcare providers must recognize that bias may exist in the data they provide to train the ML models, and they must ensure that these biases are corrected to prevent any false positives or misdiagnoses.
- *Uncontrolled data sources*: The image dataset used for ML models may come from multiple sources, which may be difficult to control for quality and accuracy.
- *Limited flexibility*: ML models have limited flexibility due to the heavy dependence on training data. The model's performance may suffer when contextual images are added to the diagnostic process.
- *Overfitting*: Overfitting occurs when an ML model is too complex and captures patterns that may not generalize, leading to inaccurate predictions on unseen data. It can lead to erroneous diagnoses when ML models are trained and tested on limited datasets.
- *Lack of Interpretability*: Because ML models aren't easy to understand, it's hard to know why a particular prediction was made. It makes it hard to trust the results and could raise ethical concerns.
- *Computational cost*: Training an ML model is computationally expensive, requiring significant computing power and time depending on the model's complexity and the dataset used for training. These costs can be too high for systems that cannot afford or do not have access to the resources needed to train these models.
- *False positives or negatives*: ML models can lead to false-negative results, meaning they can incorrectly identify a healthy person as having lung disease. In the case of a false positive, a patient with lung disease is considered a healthy individual. It could happen because of imperfect training data that does not accurately reflect the behavior of the disease or due to misclassification in the dataset being used.
- *Unreliable model performance metrics*: Due to the complexity and variability of features, it is hard to accurately assess or measure how well an ML model works when diagnosing a disease.

### Observed concerns about imaging modalities

The researchers investigated a variety of imaging modalities; Table 12 provides an overview of the various imaging modalities examined. Table 12 makes it relatively easy to comprehend that X-rays and CT scans have surpassed all other imaging methods like PET, MRI, and other imaging modalities. The diagnosis of prominent lung ailments through primary imaging modalities is as presented:

**Table 12:** Machine learning and sub-fields

Imaging Modality Type	Article Investigated
X-Ray	[3, 6, 13–17, 19, 41–62, 85, 103, 137, 138, 140–143, 145, 146, 148–151, 157, 160, 163, 168–178]
CT scan	[6, 57, 59, 64–80, 82, 103, 139, 147, 152–156, 158, 159, 161, 162, 164, 166, 167, 178–181]
PET	[6, 68, 69, 73, 81–103]
MRI	[7, 83–85, 165]
Infrared	[144]
SSMI	[8, 86–88]
Molecular	[9]
At-bedside	[89]

#### Pneumonia

Pneumonia can be detected through various imaging modalities, including X-ray, CT, PET, and MRI. X-rays can detect the presence of pneumonia by looking for areas of increased density in the lungs.

These areas are caused by fluid or inflammation and can be seen as white patches on the X-ray. X-rays are the most commonly used imaging modality for diagnosing pneumonia. CT scans provide a more detailed view of the lungs than X-rays and can detect subtler signs of pneumonia, such as small pockets of fluid or inflammation. PET can be used to detect the presence of pneumonia. It works by injecting a radioactive tracer into the body and scanning it with a special camera. The images produced can help doctors identify areas of inflammation and fluid accumulation in the lungs, which are common pneumonia symptoms. PET scans are beneficial for diagnosing complicated cases where other imaging techniques, such as X-rays or CT scans, may be inconclusive. PET scans can also help to differentiate between bacterial and viral forms of pneumonia. MRI is used less often to detect pneumonia, but it can provide a detailed image of the lungs and other organs in the chest.

#### Lung cancer

Lung cancer can be detected using X-ray images. An X-ray can reveal abnormal masses or nodules that may indicate a tumor or other abnormality. Further testing, such as a CT scan, may be preferred to confirm the diagnosis if an anomaly is found. CT scans are the most commonly used imaging modality. They can provide detailed images of the lungs, which can be used to identify tumors due to their ability to detect large and small nodules, enlarged lymph nodes, and other suspicious areas. PET scans are also used to detect cancer by detecting changes in cellular metabolism that occur with certain cancers. PET scans are often used along with CT scans to provide more detailed information about a tumor's size, shape, and location. MRI is often used to assess cancer's spread, or metastasis, from its primary site.

#### COVID-19

COVID-19 detection can be done using X-rays, CT scans, and MRI scans. X-ray is the most commonly used imaging modality for COVID-19 detection as it provides good image quality to detect pneumonia, one of the most common symptoms associated with COVID-19. CT scans provide more detailed images of the lungs than X-rays and can help detect other lung abnormalities associated with COVID-19, such as ground glass opacities or consolidations. It is also possible to see COVID-19 using PET-CT images. PET-CT images can show areas of increased metabolic activity that could indicate an infection. MRI scans are not commonly used for COVID-19 detection because they produce lower-resolution images than CT scans. Conclusively, a chest X-ray is the easily accessible and most common imaging modality used to diagnose lung diseases. A CT scan can provide more detailed images of the lungs than a chest X-ray and help identify subtler signs, such as small areas of infection or inflammation. They are the ones that researchers prefer to employ while doing research.

### Observed concerns about datasets

Image datasets are necessary for the development of computer vision and ML models. They provide a source of input data to train, validate, and test an ML model. Access to large datasets is necessary to develop ML models that accurately identify lung disease in images. Image datasets are the backbone of any ML model and play a significant role in its success. In addition, publicly accessible image datasets provide insights, helping researchers develop automated ML models. An overview of the numerous imaging datasets on lung diseases is presented in Table 13.

**Table 13:** Numerous imaging datasets explored relevant to prominent lung diseases

Lung Disease Type	Imaging Modality	Imaging Dataset Explored
Pneumonia	X-Ray	[42–55, 137, 138, 140–143, 145, 146, 148–151]
	CT scan	[63, 147, 152, 153]
	Infrared Thermal Imaging	[144]
Lung Cancer	X-Ray	[44, 46, 52, 56, 157, 160, 163]
	CT scan	[64–73, 154–156, 158, 159, 161, 162, 164]
	PET	[68, 69, 73]
	MRI	[165]
COVID-19	X-Ray	[47, 48, 53–55, 57–60, 168–178]
	CT scan	[57, 59, 74–78, 178–181]

The imaging datasets employed by researchers in their investigations were maximally proposed or constructed, and they were sometimes given names such as COVIDX [168], COVID-R [175], and COVQU [176]. Researchers also utilized and prioritized publicly available datasets, such as LIDC/IDRI [154–156], JSRT [157], NLST [161], and several others, in their research.

It demonstrates conclusively that X-rays and CT scans outperform other imaging datasets. It has also been discovered that in the detection of pneumonia, X-ray datasets are preferred most of the time; in the detection of lung cancer, CT scan datasets are primarily selected; and in the detection of COVID-19, X-ray datasets are preferred first, followed by CT scan datasets.

### Observed concerns about ML

Table 14 shows that standard ML, DL, CNN, transfer learning, and ensemble learning algorithms can definitively evaluate lung imaging modalities such as X-ray, CT scan, MRI, and infrared thermal imaging to detect pneumonia, lung cancer, and COVID-19. When diagnosing pneumonia, as laid out in Table 14, it is simple to observe that the automatic detection and classification of pneumonia in chest X-rays are primarily accurate and attainable with DL-based approaches such as CNNs. Compared to traditional ML procedures, this one is more reliable and gives a faster and more precise diagnosis. The diagnosis also relies on transfer learning to be reliable. In combination with CNN, transfer learning and ensemble learning also support the analysis of X-rays. CT scans are used for diagnosis in ML and its sub-fields; however, they are less recommended than X-rays since an X-ray is adequate for diagnosing pneumonia.

**Table 14:** Numerous machine learning and sub-field in lung disease diagnosis

Lung Disease	Imaging Modality	ML/Sub-domains	Article Investigated
Pneumonia	X-Ray	Conventional ML	[145]
		DL/CNN	[140–142, 146, 148–151]
		Ensemble Methods	[143, 148]
		Transfer Learning	[143, 146, 148, 150]
	CT scan	Conventional ML	[152]
		DL/CNN	[147, 153]
		Ensemble Methods	[152]
		Transfer Learning	[153]
	Infrared Thermal	Conventional ML	[144]
Lung Cancer	X-Ray	Conventional ML	X
		DL/CNN	[157, 160, 163]
		Ensemble Methods	X
		Transfer Learning	[157]
	CT scan	Conventional ML	[155, 156, 159, 166, 167]
		DL/CNN	[154, 158, 159, 161, 162, 164]
		Ensemble Methods	[155]
		Transfer Learning	X
	MRI	Conventional ML	[165]
COVID-19	X-Ray	Conventional ML	[170, 171, 178]
		DL/CNN	[168–178]
		Ensemble Methods	[171, 174]
		Transfer Learning	[170, 171, 177]
	CT scan	Conventional ML	[178]
		DL/CNN	[178–181]
		Ensemble Methods	X
		Transfer Learning	[180, 181]

Employing CNNs to analyze CT images successfully identifies and categorizes lung nodules, which are minor growths that may signify lung cancer. CNNs can be trained on massive CT scan data to learn the features associated with various lung nodules, allowing for reliable identification and classification. CNN has been used in many studies to accurately identify lung nodules, making it a viable technique for the early identification of lung cancer. Conventional ML is preferred in tandem with CT scans as well. The necessity for qualitatively crisper imaging, provided by a CT scan, makes X-rays a less likely option than they would otherwise be. It is also observed that transfer learning and ensemble learning are less preferred in diagnosing lung cancer, which can be easily observed in Table 14.

Training a CNN on X-ray images makes identifying the COVID-19-typical pattern of pulmonary in-filtrates feasible. Multiple research studies have previously demonstrated that this method is effective, indicating that CNNs can accurately identify COVID-19. When using DL-based techniques like CNNs, X-rays come out on top as the preferred imaging method. It has been discovered that CNN is more accurate than the conventional ML approaches. Transfer learning and ensemble learning are also utilized with ML and CNN. CNN is preferable over all other ML methods when considering CT scans.

It is also observed that the introduced novel method has a greater dominance over existing ML and DL methods put forth by researchers.

### ML pathway

ML methods can spot patterns in medical imaging that may indicate the presence of lung disease. Prominent lung diseases can be diagnosed using ML models, with the classification being based on the features. ML-based methods are increasingly being used to detect and diagnose significant lung diseases. Large datasets of images are used to train ML algorithms to detect lung abnormalities. The algorithm is then evaluated on new images, where it can recognize and categorize various forms of lung irregularities. In particular, DL models based on CNNs have been developed and employed for detecting various lung abnormalities through medical imaging.

The solution to all the issues included an explanation and observations made throughout the review. It is observed that most of the research follows the pathway of ML:

- *Image Acquisition*: Researchers amassed vast and varied images from chest X-rays, CT scans, and other imaging modalities associated with certain lung diseases [6–9]. These images have been labeled chiefly for identification purposes, mostly. Most researchers preferred publicly accessible datasets in comparison to private datasets [42–55, 63, 137, 138].
- *Image Preprocessing*: Researchers preprocessed the image dataset to reduce noise and outliers and normalize the data for superior results. Significant preprocessing operations had been carried out, such as the selection and modification of attributes, the imputing of missing values, the normalization of features, and the elimination of noise. The images are preprocessed to reduce their dimensionality. They converted images into numerical data by breaking them into individual pixel colors to input them into the ML model. Once the preprocessing is completed, the dataset is generally split into training and test datasets so that each portion adequately represents relevant cases [19, 140–167].
- *Feature Extraction and Relevant Feature Selection*: Researchers extracted image features, such as edges, shapes, and textures, and selected relevant features so that ML algorithms could assess them [151–181].
- *Training of the ML Model*: Researchers trained the ML model using labeled datasets with known outcomes to detect patterns associated with the specified disease class in supervised learning. In the case of unsupervised learning, the ML model can also draw a pattern and identify the disease with the unlabeled data. They chose an appropriate model and algorithm to learn from the input dataset. With CNN, they trained the model on processed data with different learning rates and weights or different architectures to find the best performance [121–125, 128].
- *Performance Metrics*: Researchers evaluated the ML model using a particular performance metric. Evaluate by measuring performance metrics on how well it learned from the training data. After training the model, it is evaluated using metrics such as accuracy, recall, precision, F1 score, etc., which measure how well it performs on unseen data samples. In DL and CNN, monitoring accuracy and other metrics such as sensitivity and specificity is performed after each training epoch to ensure all parameters are fine-tuned and that training ends with an acceptable performance score that has attained desirable precision and recall scores [140–181].
- *Evaluation*: The ML model was applied to fresh datasets by the researchers so that they could make predictions about the results of their research studies or identify cases of lung disease [140–181].

### Observed concerns about performance metric

Researchers chose the accuracy performance metric as the primary metric because it was more important than the other metrics used to evaluate the model. Because of this, this review focused on this metric and gave an overview of it for each prominent lung disease. Accuracy is the most notable performance metric since it measures performance consistently across all classes. Since all misclassified samples are assigned the same value, accuracy can better detect slight performance discrepancies.

#### Analysis of performance metrics for pneumonia diagnosis

When it came to the diagnosis of pneumonia, most of the researchers calculated several types of performance metrics; nonetheless, accuracy was the metric most highly esteemed and presented in Table 15. One solitary study [141] did not achieve this since the researcher's work was not executed as desired, but all other investigations did.

**Table 15:** Accuracy of pneumonia diagnosis research

Performance Metric	Article Investigated
Accuracy	[140, 142–153]
Others	[141]

#### Analysis of performance metrics for lung cancer diagnosis

In lung cancer diagnosis, most researchers computed different kinds of performance metrics, but accuracy was the most preferred metric, as presented in Table 16. The investigations [161] and [166] were the only ones that did not favor this since other metrics required more relevance than accuracy.

**Table 16:** Accuracy of lung cancer diagnosis research

Performance Metric	Article Investigated
Accuracy	[154–160, 162–165, 167]
Others	[161, 166]

#### Analysis of performance metrics for COVID-19 diagnosis

As we observed in the trend analysis of COVID-19, in which we analyzed the meteoric increase of searches for COVID-19, the spontaneous growth of research conducted on COVID-19 is tremendous. It's something that we noticed in our investigation of the trend of COVID-19. The investigators in the COVID-19 study generally prioritized accuracy as a critical performance criterion, except for [179]. Table 17 presents the accuracy of COVID-19 diagnosis research.

**Table 17:** Accuracy of COVID-19 diagnosis research

Performance Metric	Article Investigated
Accuracy	[168–178, 180, 181]
Others	[179]

## Conclusion

The investigation highlights the intricacy of identifying prevalent pulmonary conditions, including COVID-19, pneumonia, and lung cancer, emphasizing the critical importance of advanced ML and imaging diagnostic techniques. The imaging datasets made available to the public underscored the significance of segregating data according to disease specifications because each prominent lung disease has symptoms that specific imaging modalities can detect because of their unique properties. The research demonstrates the inclination towards X-rays as the prevailing imaging modality, owing to their widespread availability and usage. CT scans are considered a secondary option, offering improved detail. ML techniques, particularly CNNs, transfer learning, and ensemble learning, have been crucial in speeding up and enhancing the accuracy of diagnoses. These approaches use computed imaging parameters to classify data automatically. The research contributes substantially by examining significant lung disorders, analyzing relevant datasets, and thoroughly evaluating ML methods. It also highlights the difficulties involved and suggests some solutions. The methodical exploration focuses on methodologies used in published results and provides significant perspectives for researchers in this field. Although the observations contribute significantly, it is crucial to recognize critical limitations. The use of publically available datasets may have biases, and the ability of ML models to apply to various populations has to be further investigated. The research focuses on specific imaging techniques and does not incorporate upcoming technology. Furthermore, it is crucial to focus on the comprehensibility of ML models when applied to clinical decision-making. To further advance the study, Investigating the incorporation of multi-modal datasets and real-time ML applications in healthcare environments might be advantageous. Furthermore, alternate imaging techniques, as opposed to the ones now being investigated, might enhance the comprehensiveness. Moreover, adopting ML-based diagnostic tools might facilitate the appropriate use of these technologies in the healthcare sector.

## Data Availability

The images, data, and datasets presented and analyzed during the review are available in the publicly available repositories and require no permissions. These images, data sets, and datasets came from public domain sources and were adequately cited and referenced in the manuscript.

## Abbreviations

AFB: Acid-Fast Bacteria
ANN: Artificial Neural Network
ARDs: Acute Respiratory Distress Syndrome
AI: Artificial Intelligence
AP: Anterior-Posterior
CDR: Chronic Respiratory Disease
CART: Classification and Regression Tree
CT: Computer Tomography
CLAHE: Contrast Limited Adaptive Histogram Equalization
CNN: Convolutional Neural Network
CXR: Chest X-Ray
DL: Deep Learning
DNN: Deep Neural Network
EIT: Electrical Impedance Tomography
EU: European Union
FDG: Fluorodeoxyglucose
GAN: Generative Adversarial Network
GDPR: General Data Protection Regulation
GA: Genetic Algorithm
GUI: Graphical User Interface
GLCM: Gray Level Co-Occurrence Matrices
GWO: Gray Wolf Optimization
ICFA: Improvised Cuttlefish Algorithm
ICSA: Improvised Crow Search Algorithm
IGWA: Improvised Grey Wolf Algorithm
ILD: Interstitial Lung Disease
LDA: Linear Discriminant Analysis
LBP: Local Binary Pattern
LUS: Lung Ultrasonography
ML: Machine Learning
MARS: Multivariate Adaptive Regression Splines
MRI: Magnetic Resonance Imaging
MLP: Multilayer Perceptron
NSCLC: Non-Small-Cell Lung Cancer
PET: Positron Emission Tomography
PRISMA: Preferred Reporting Items for Systematic Reviews and Meta-Analyses
PCA: Principal Component Analysis
RT-PCR: Real-Time Reverse Transcriptase Polymerase Chain Reaction
RELU: Rectified Linear Unit
RNN: Recurrent Neural Network
ROI: Regions of Interest
SCLC: Small-Cell Lung Cancer
SPECT: Single Photon Emission Computed Tomography
SSMI: Sputum Smear Microscopy Image
SVM: Support Vector Machine
WHO: World Health Organization
